# Low levels of salivary metals, oral microbiome composition and dental decay

**DOI:** 10.1038/s41598-020-71495-9

**Published:** 2020-09-04

**Authors:** Elyse Davis, Kelly M. Bakulski, Jaclyn M. Goodrich, Karen E. Peterson, Mary L. Marazita, Betsy Foxman

**Affiliations:** 1 Center for Molecular and Clinical Epidemiology of Infectious Diseases, University of Michigan School of Public Health, 1415 Washington Heights, Ann Arbor, MI 48109-2029 USA; 2 Department of Epidemiology, University of Michigan School of Public Health, 1415 Washington Heights, Ann Arbor, MI 48109 USA; 3 Department of Environmental Health Sciences, University of Michigan School of Public Health, 1415 Washington Heights, Ann Arbor, MI 48109 USA; 4 Department of Nutritional Sciences, University of Michigan School of Public Health, 1415 Washington Heights, Ann Arbor, MI 48109 USA; 5 Department of Oral Biology, Center for Craniofacial and Dental Genetics, School of Dental Medicine, University of Pittsburgh, Pittsburgh, PA 15219 USA; 6 Department of Human Genetics, Graduate School of Public Health, University of Pittsburgh, Pittsburgh, PA 15219 USA; 7 Clinical and Translational Sciences Institute, School of Medicine, University of Pittsburgh, Pittsburgh, PA 15219 USA

**Keywords:** Microbiome, Dental caries, Environmental impact

## Abstract

Salivary microbiome composition can change following exposure to environmental toxicants, e.g., heavy metals. We hypothesized that levels of salivary nutrients and metals would correlate with salivary microbiome composition and be associated with dental decay. Here we assess the salivary concentrations of 5 essential minerals (cobalt, copper, manganese, molybdenum, and zinc), 4 metals with some evidence of normal physiological function (chromium, nickel, tungsten, and vanadium), and 12 with known toxicity (antimony, arsenic, barium, beryllium, cadmium, cesium, lead, mercury, platinum, thallium, tin, and uranium), and their associations with salivary microbiome composition and dental decay in 61 children and adults. 16 metals were detected in 54% of participants; 8 were found in all. Marked differences in salivary bacterial taxa were associated with levels of antimony, arsenic, and mercury, after adjusting for multiple testing. Further, antimony levels were associated with the presence of decayed teeth. Thus, salivary metal levels, even at low concentrations, may impact oral health.

## Introduction

The salivary microbiome provides a window into the overall health of the body and likely changes in response to environmental exposures, such as metals. Like humans, bacteria require certain metals to carry out metabolic processes, and often compete with host cells to take up these metals through transporters and other acquisition systems^1,2^. However, toxicity is a concern for bacteria, as some metals—such as cadmium and copper—are bactericidal at certain concentrations^3^. Further, even essential metals can be toxic, depending on the dose (Supplemental Tables S1–S3). Therefore, we hypothesized that there will be changes in the salivary microbiome composition in response to salivary metal levels which may directly affect oral health. Dental caries is the most common chronic disease in children aged 6–19^4^ and periodontal disease is the most common cause of adult tooth loss^5^. The oral microbiome is an important determinant of both dental caries and periodontal disease. As it is now clear that the microbiome at all body sites responds to environmental stressors such as pathogens and environmental chemicals, it is critical to understand the implications of metal exposures on the structure, composition, and function of the oral microbiome, taking into account whether the metal is exclusively toxic, an essential mineral, or has some physiological functions.

When certain toxic metals, e.g., lead or cadmium, are measured in blood or urine the resulting levels can be compared to guidelines for concentrations of concern^6^; however, no guidelines exist for salivary metal levels. Saliva biomarker exposure assessment could be particularly useful in studies where the oral cavity is the primary target region of toxicity (e.g., studies of the oral microbiome or dental health). Some studies have assessed salivary metals, but the number of publications and the scope of metals assessed are limited. Lead^7,8^ and mercury^9^ have been of particular interest, but there are some reports on salivary iron, magnesium, and zinc^10^.

Two major gaps in the literature motivated us to quantify salivary concentrations of 21 metals among 61 healthy individuals, to describe associations between salivary essential mineral and metal levels with composition of the salivary microbiome, and describe the associations between metal levels and dental decay. First, lack of information on how metals affect the oral bacterial community, and second, the lack of a comprehensive study of multiple metals in saliva and their impact on the oral microbiome, and third, limited studies of the associations of metal levels and the presence of dental decay.

## Results

The 61 participants included children (21% aged 7–17) and adults (79% aged 18–45) from 37 families (an average of 2 participants per family, range of 1–4). The average age was 26.8 years (SD: 9.39 years); 60% were female; 43% were from West Virginia and the remainder from Pennsylvania in the United States. About one-quarter (24.6%) of participants had an annual income less than $10,000, and 38.6% earned between $10,000 and $35,000 per year. A history of dental decay was common: 98% of adults and 69% of children had one or more decayed, missing or filled teeth, and 75% of adults and 31% of children had an untreated dental lesion.

### Metals were detected in all saliva samples

The minimum limit of detection (LOD) for most salivary minerals and metals was between 0.1 and 0.5 parts per billion (ppb), with zinc having the highest LOD at 30 ppb. Table 1 shows detection limits and detected distributions for each metal. The percentage of participants with concentrations above the LOD for each metal ranged from 7 to 100%. 16 of the 21 metals had a detection rate of 54% or higher. The essential minerals and those with some evidence of normal physiological functions were detected in almost all participants (76–100%). Four of the 12 heavy metals were detected in only a small proportion of participants: thallium, beryllium, platinum, and uranium (7%, 10%, 5%, and 20%, respectively).

**Table 1:** Metals detected in the saliva, limit of detection (LOD) and distribution.

Metal	Salivary metal tertiles (ppb)					
	LOD	Minimum	33rd percentile	Median	66th percentile	Maximum
**Essential**						
Cobalt	0.1	0.23	0.42	0.55	0.64	1.87
Copper	10	< LOD	27.42	37.28	52.79	394.79
Manganese	5	< LOD	10.74	14.46	21.76	94.37
Molybdenum	0.2	0.24	0.82	0.95	1.20	4.49
Zinc	30	97.81	265.72	303.69	382.77	10,198.95
**Other heavy metals**						
Antimony	0.1	0.16	2.43	2.73	3.40	13.33
Arsenic	0.2	< LOD	< LOD	0.14	0.25	2.65
Barium	0.5	2.17	5.58	15.71	29.24	69.86
Beryllium	0.5	< LOD	< LOD	< LOD	< LOD	2.49
Cadmium	0.5	< LOD	0.30	0.49	0.58	7.16
Cesium	0.2	< LOD	0.44	0.50	0.66	1.98
Lead	0.1	0.82	2.64	4.12	7.25	252.07
Mercury	0.1	< LOD	< LOD	0.33	1.03	446.90
Platinum	0.1	< LOD	< LOD	< LOD	< LOD	0.29
Thallium	0.5	< LOD	< LOD	< LOD	< LOD	1.74
Tin	0.5	< LOD	0.77	1.62	3.08	195.03
Uranium	0.1	< LOD	< LOD	< LOD	< LOD	0.51
**Some evidence of physiological function**						
Chromium	0.2	0.73	1.49	1.81	2.26	10.78
Nickel	2	3.306	6.63	7.83	8.71	26.07
Tungsten	0.1	< LOD	0.11	0.20	0.25	19.34
Vanadium	0.1	< LOD	0.20	0.36	0.41	1.60

Stimulated saliva samples were obtained from 13 children age 7–17 and 48 adults aged 18–45 individuals from the Center for Oral Health Research in Appalachia Study I.

We observed differences in salivary levels for some metals by age and income but none by sex. Using the Wilcoxon rank-sum test, there was a significantly higher median level of cesium among adults (0.55 ppb) than among children (0.34 ppb) (p = 0.02), but no other metals. Levels of copper were higher among those with incomes in the $10,000–35,000 range compared to those with < $10,000 or those > $35,000 (Kruskal–Wallis p = 0.04) (Supplementary Table S5).

### Salivary metals were not associated with diversity of the salivary microbiome, but were associated with microbiome composition

After sequencing the V6 region of the 16S rRNA gene in saliva samples, we identified 134 different bacterial species across all samples [range 128–134; median ~  = average 133 (SD = 1.2)]**;** the average number of species did not differ by age, sex, or income. With one exception (barium), there were no significant differences in alpha diversity of the oral microbiome and tertile of metal exposure (assessed using Shannon and Chao1; Table S4). After rarefication, there were no significant differences in diversity using Shannon or Chao1 measures.

For each metal and bacterial taxa pair, we next compared the relative abundance among participants in the highest tertile of metal level to those in the lower two metal tertiles using ALDEx2^11^. After Benjamini–Hochberg correction, the relative abundance of one or more bacterial taxa varied by exposure level for three metals: antimony, arsenic, and mercury (Table 2). The relative abundance of four unclassified Lactobacilli, and *Escherichia coli* were higher among those in the highest tertile of antimony (Fig. S1). Since the four unclassified Lactobacilli could not be identified to the species level using the CORE database, we searched the NCBI Basic Local Alignment Search Tool for possible identification^12^. The species with the highest query coverage, E.value, and percent identity for these Lactobacilli are listed in Supplemental Table S7. It is likely all 4 unidentified Lactobacilli associated with levels of antimony are *Lactobacillus acidipscis, although*, *Lactobacillus pobuzihii* and *Lactobacillus salitolerans* are also possible for *Lactobacillus_sp*_11798 and *Lactobacillus_sp*_11808. While not statistically significant, higher levels of antimony were associated with a lower relative abundance of Granulicatella. Arsenic was associated with a significant decrease in species within the genus Prevotella. Mercury was associated with significant decreases in species within the genera Neisseria, Granulicatella and Abotrophia, and significant increases in *Streptococcus species* and *Prevotella species*.

**Table 2:** Effect sizes from ALDEx2 analysis.

Taxa	Antimony	Arsenic	Mercury
*Abiotrophia_defectiva*	− 0.10	0.16	− **0.52**
*Escherichia_coli*	**0.69**	0.07	− 0.03
*Granulicatella_elegans*	− 0.46	0.15	− **0.46**
*Lactobacillus_sp_11798*	**0.67**	0.08	0.04
*Lactobacillus_sp_11800*	**0.66**	0.03	0.02
*Lactobacillus_sp_11808*	**0.69**	0.07	0.01
*Lactobacillus_sp_11809*	**0.65**	0.05	0.06
*Neisseria_meningitidis polysaccharea*	− 0.05	0.12	− **0.45**
*Prevotella_pleuritidis*	0.21	− **0.70**	− 0.03
*Prevotella_sp_9409*	− 0.04	− 0.10	**0.49**
*Prevotella_sp_9421*	− 0.05	− 0.11	**0.54**
*Streptococcus_GU045364*	0.04	0.08	**0.33**

Stimulated saliva samples were collected from 61 individuals from the Center for Oral Health Research in Appalachia Study.

Antimony was significantly associated with increases in relative abundance of several species of acid-producing bacteria of the genus *Lactobacillus*. Associations with toxic metals arsenic and mercury are shown for comparison. The effect size comparing those in the highest tertile to the lower two tertiles for each species is the difference between groups relative to an estimate of within-group dispersion. Only associations with Benjamini–Hochberg corrected p-values < 0.1 are shown, with significant values in bold. The effect sizes are displayed within the cells; increases in taxa abundance associated with increases in metal level are positive. Several oligotypes of *Lactobacillus* and *Prevotella* were identified that could not be resolved to species level; these are indicated by a number.

The relative abundance of 66% of the taxa differed by age using the ALDEX2 method (Supplemental Table S8). This was true for all but three of the species that were significant for one or more metals (*Lactobacillus_sp_11808*, *Prevotella pleuritidis*, and *Escherichia coli).*

As metal exposure and risk of dental caries are likely correlated within families, we fit linear mixed models predicting the centered-log ratio transformed species counts for the taxa that were found to be statistically significant with levels of antimony in the ALDEx2 analyses that adjusted for correlations within families. Consistent with ALDEx2 analyses, there was increase in each taxa abundance with higher levels of antimony. For comparison, we also fit models predicting the centered-log ratio transformed species counts of selected species associated with oral health. Again, the direction of the model estimates were consistent with the unadjusted associations obtained using ALDEx2 (Supplemental Table S6).

### Several metals are associated with presence of an untreated dental lesion

**Figure 1:**
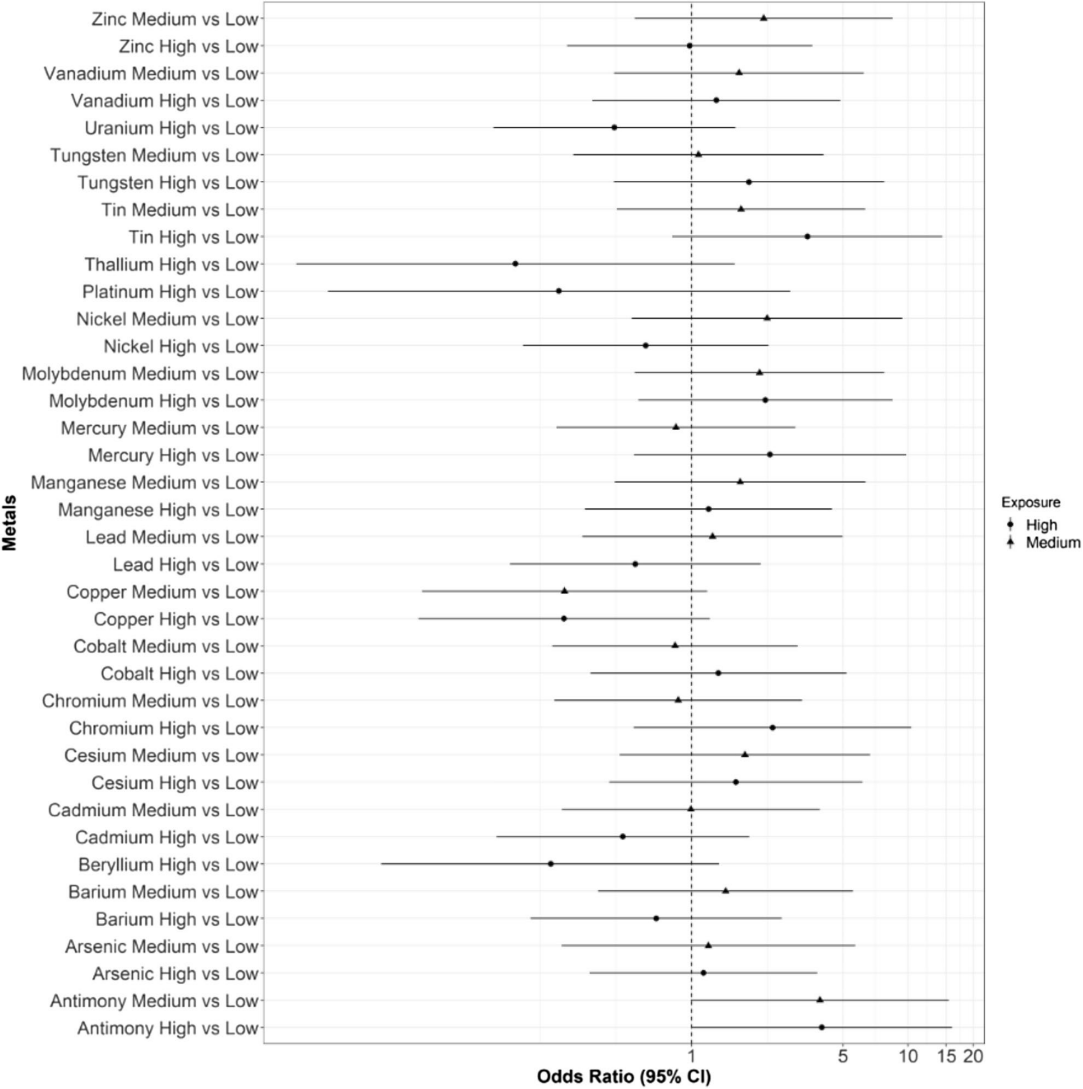
Adjusted odds ratios for presence of decayed teeth by selected heavy metal tertile (high versus low (circles), medium versus low (triangles). X axis is shown on a log scale. Saliva samples were collected from 61 individuals from the Center for Oral Health Research in Appalachia Study.

We used logistic regression models to calculate the odds of dental decay, here defined as an untreated carious lesion, comparing the upper to the lowest and middle to the lowest tertile of metal exposure, adjusted for participant age (Fig. 1). Although the confidence intervals of the odds ratios overlap one, the results suggest an association between several metals and presence of an untreated dental lesion. Notably, there was an association with salivary levels of antimony in the upper (OR = 3.91; 95% CI 0.99, 15.40) and medium (OR = 3.99; 95% CI 1.00, 15.94) tertiles compared to the lowest tertile of exposure. As the presence of an untreated dental lesion is associated with income (which is also associated with other correlates of a dental lesion, e.g., diet and access to care), we repeated these analyses including income as a covariate. The point estimates were similar in direction and magnitude, although, as expected the confidence limits were wider (Supplementary Table S9). We also considered whether there were differences in taxa by income, and there were no taxa whose relative abundance was statistically significant by income (comparing income groups < $10,000; $10,000-$35,000; and + $35,000, using Kruskal–wallis testing with ALDEx2) after Benjamini–Hochberg correction.

## Discussion

To our knowledge, ours is the first study to comprehensively measure salivary concentrations of 21 essential minerals and metals in healthy children and adults, and to compare salivary concentrations to the composition of the oral microbiome. We add to the limited published information on salivary concentrations for lead, copper, manganese, zinc, cadmium, and mercury^7–10^ in various study populations, and measure an additional 15 essential minerals and metals in human saliva for the first time. The inductively coupled plasma mass spectrometry (ICP-MS) analysis allowed us to detect these metals at very low concentrations, and detection was observed in most individuals. We found novel associations between high levels of three metals (arsenic, antimony, and mercury) and the relative abundance of selected bacteria in the saliva, and between antimony and presence of an untreated dental lesion. Antimony is commonly found in flame- retardant materials, paint, enamels, glass and pottery. It is also an alloy used in the production of batteries and bullets. Although dietary exposure to antimony is low, it may be found in drinking water (Supplementary Table S2).

The salivary metal concentrations we observed for lead, copper, manganese, zinc, cadmium, and mercury are generally consistent with those reported in previous studies. A study among 29 healthy Polish adults who did not smoke, use dietary supplements or have known metal exposure, used plasma induction for salivary metal detection of some of the same metals^10^. Mean concentrations of cadmium, copper, manganese, and lead were similar to that reported here but we observed higher levels of zinc (958.87 ± 1988.22 ppb in our study vs. 75.3 ± 74.4 ppb)^10^. Our mean salivary lead concentrations were also somewhat higher than that reported in a low-income population of 969 Michigan African Americans where the mean salivary lead levels were 2.4 ± 0.13 ppb, respectively^7^ compared to our overall mean of 14.94 ± 38.5 ppb. These differences might reflect difference in collection—the Polish and Michigan studies used unstimulated saliva collected following a rinse with sterile water and we used stimulated saliva, or differences in exposure by study population. Our study contributes to an emerging field of salivary metals exposure assessment by providing consistent measurement ranges to previously documented metals and benchmarking ranges for newly measured metals.

Our observed associations between microbiome taxa and arsenic and mercury probably are due to their known toxicity at low doses and in multiple systems. Further, mercury is used in dental amalgams, so we might expect those with multiple fillings to be more likely to have an untreated dental lesion. In a murine model, zinc deficiency and arsenic exposure individually resulted in changes in the diversity of the gut microbiome and together acted synergistically^13^. This is potentially relevant as the oral and gut microbiomes are predictive of each other^14^. However, our sample size was too small to evaluate this association.

This is the first report of an association between antimony with microbiome composition and an untreated dental lesion. The two findings together suggest that the association might be important, as several species of acid-producing genus *Lactobacillus* increased in relative abundance in the presence of antimony. The *Lactobacilli* are acid producers, and have long been associated with dental decay^15^. Additionally, *Granulicatella*—whose relative abundance has been negatively associated with dental caries^16^—decreased in abundance with higher levels of antimony. A search for microbiome studies of antimony found only 14 studies (all in soil or water)^17–30^.

Findings from this study should be considered in light of limitations. A relatively small sample size limited our ability to simultaneously adjust for confounding effects of age, income and history of dental decay, that may be associated with metal exposure, oral microbiome composition and/or presence of an untreated dental lesion. Additionally, our methodology did not allow us to distinguish intracellular metals from those free-floating in saliva. Further, although the salivary microbiome overlaps with that of dental plaque^31^, we might have gotten stronger signals if we had measured dental plaque microbiome which are directly involved in cariogenesis. While these limitations should be taken into account when generalizing to other populations, they do not invalidate our major findings that low levels of many metals are detectable in the saliva, and that these levels are associated with the relative abundance of bacteria present in the saliva that are in turn associated with diseases of the oral cavity.

Saliva has the potential to serve as a minimally invasive biomarker of metal exposures, especially in studies focused on oral health outcomes. For example, in studies focused on the oral microbiome or dental health as the primary outcome of interest, quantifying metals in the saliva is advantageous as these are the concentrations directly in contact with the target site of toxicity. Furthermore, saliva collection may be more feasible than urine or blood collection for epidemiological studies recruiting in remote locations (e.g., household visits in rural areas without laboratory facilities) or recruiting sensitive populations (e.g., young children). In current exposure science, some biological specimens are considered better biomarkers of metal exposure than others, such as whole blood for lead^32,33^ serum for copper and zinc^34^, and urine for chronic exposure to arsenic, cadmium, chromium, or inorganic mercury^35–38^. For other metals, there is less of a consensus as to whether blood, serum, urine, hair, or nails serve as better biomarkers, and whether the exposure is chronic or acute also impacts interpretation of biomarker concentrations. Future studies should explore the broader application of easily-obtainable saliva as a biomarker by profiling salivary metal concentrations in larger populations with known sources of exposure and explore the associations with oral health.

Bacteria present in the human microbiome, such as *Escherichia coli*, can reproduce as frequently as every 20 min under optimal conditions^39^, and rapidly respond to environmental exposures of all kinds—including to metals. This property might be leveraged in the future to develop diagnostics or prognostics using saliva. Further, microbiota alter the body’s ability to absorb and metabolize environmental exposures^40^. Motivation for continued research on the salivary microbiome is supported by the results we observed, as well as a previous study describing an association between chronic arsenic exposure and an increase in dental caries amongst children^41^. Dental caries remains a major public health problem: in the US 12.4 million children and 37.6 million adults have untreated caries, with substantial disparities by race and income^42^. The ability to measure metals and microbes in a single sample is a great strength, as it eliminates the need to extrapolate using data from other collection sites.

## Methods

### Study population and sample collection

The Center for Oral Health Research in Appalachia (COHRA) is an oral health initiative in the Appalachian region of Pennsylvania and West Virginia, focused on studying oral health disparities. The COHRA1 dataset includes families with at least one biological child < 18 years of age were recruited from four counties deemed representative of rural Appalachia. In person interviews were used to obtain demographic characteristics, and qualified dentists or hygienists conducted a dental examination, physical evaluation, and microbiological and salivary sampling. The full collection protocol for this cross-sectional study was published previously^43^. Approval was obtained from the Institutional Review Boards at the University of Pittsburgh and West Virginia University, and all relevant guidelines and regulations were adhered to during the duration of the study. Informed consent was obtained from all adult participants upon enrollment. The consent of a legal parent or guardian was obtained for all individuals with less than 18 years of age. Below we describe details for saliva exposure and microbial biosampling that occurred between 2006 and 2009, in a previous study led by co-author Dr. M.L. Marazita^44^.

At the dental assessment by calibrated dental professionals, stimulated saliva was collected by having participants chew on a paraffin pellet for one minute. Participants were instructed not to eat, drink, or brush teeth at least two hours before screening and assessments were conducted about two hours into the interview. For this analysis, our outcome was the presence of one or more unfilled carious lesions^44^. Samples were stored in a cryogenic tube without preservative. Samples were frozen at − 80 °C until testing. DNA was isolated and sequenced in 2012. This pilot study used a subset of the saliva samples from a previous COHRA I publication^44^ that characterized the salivary microbiome among 173 individuals. These participants came from 49 different families, in which at least two children and at least one adult provided a saliva sample^43^. For this analysis we used all samples from individuals > 6 years of age, where there was sufficient stimulated saliva remaining for testing. This gave us a final sample size of 61 individuals.

### Microbial detection

Microbial detection was described previously^44^. Briefly, DNA extraction of saliva samples was completed using DNeasy Blood and Tissue kits and the automated QIAcube system (Qiagen; Venlo, Netherlands). To lyse microbial cell walls, prior to DNA extraction, 80 μL of an enzyme cocktail comprised of Promega cell lysis solution (Promega; Madison, WI, USA), lysozyme, mutanolysin, RNase A, and lysostaphin (Sigma; St. Louis, Mo, USA) in 22.5:4.5:1.25:1.25:1 parts, respectively, was added to 100 μL of sample before incubation at 37 °C for 30 min. Extraction was completed using manufacturer’s instructions. DNA concentration was assessed using a Nanodrop 2000C spectrophotometer (Thermo Scientific, Waltham, MA, USA) post extraction.

Thermal amplification of the V6 region of the 16 s rRNA gene was performed using eight base pair error-correcting barcodes with conserved primers using a previously published protocol^45^. 3 ng of barcoded DNA from each sample was pooled into a single 1.5 mL Eppendorf tube. 140 ng of an in-house barcoded mock community consisting of 57 species with an overrepresentation of genera common and uncommon to a healthy oral microbiome were also included in the pool. Species included in the mock community were *Lactobacillus* spp. *Peptoniphilus* spp., and *Propionibacterium* spp. The University of Michigan DNA Sequencing Core performed sequencing of the pooled DNA using the Illumina HiSeq Platform.

### Taxonomic assignment

Paired end sequences were joined using fast length adjustment of short reads (FLASH)^46^. Reads were then demultiplexed and filtered using Quantitative Insights Into Microbial Ecology (QIIME) 1.9.0^47^. The combined final read count after filtering and demultiplexing was 178,682,752, with a range of 2,745–1,726,317 reads. The read count per sample range for our subset was 139,279–1,006,268 reads.

The QIIME data was prepared for oligotyping using the publicly available python script q2oligo.py^48^. We clustered our sequences into oligotypes using an iterative unsupervised Minimum Entropy Decomposition algorithm using Shannon entropy values^49^. Taxonomy for the oligotypes was assigned using the CORE database, specifically curated for the identification of bacteria found in the oral cavity^50^.

### Salivary metal measurements

In 2018, NSF International (Ann Arbor, Michigan) assessed the stored stimulated saliva samples from 61 COHRA participants to quantify concentrations of 21 metals using inductively coupled plasma mass spectrometry on a Thermo Fisher (Waltham, MA, USA) ICAPRQ spectrometer with an CETAC ASX-520 auto sampler. The protocol was adapted from a validated urinary analysis method (see Tables S1–S3). Samples with 1,000 µl of saliva were run at their existing dilution (n = 44). The remaining 28 samples that contained less than 1,000 µl were supplemented with deionized water until the targeted volume was attained; detected metal levels were adjusted to take into account any dilution factors. Standards with known purity and identity were used to set calibration curves and for quality control purposes. The percent nominal concentration across standards of known concentration ranged from 94 to 106%. The level of detection (LOD) was determined by running a dilution matrix blank 10 times and calculating the standard deviation of the instrument response. The LOD was defined by calculating three times the standard deviation of the instrument response. We considered cobalt, copper, manganese, molybdenum, and zinc metals as essential minerals, antimony, arsenic, barium, beryllium, cadmium, cesium, lead, mercury, platinum, thallium, tin, and uranium metals as other heavy metals, and chromium, nickel, tungsten, and vanadium metals as those with some evidence of known physiological function. The following metals were detected in less than 30% of the study population, and were excluded from the ALDEx2 analyses (metal (prevalence)): thallium (7%), beryllium (10%), platinum (5%), uranium (20%).

### Statistical analysis

Statistical analyses were performed using R statistical software (version 3.4.0)^51^ and SAS version 9.4^52^. Metal concentrations below the LOD specified in Table 1 were assigned a level of $\frac{LOD}{\sqrt{2}}$. One individual had an extremely high value for mercury (446.90 ppb) and a second individual had an extremely high value for lead (252.07 ppb). To formally test for extreme outliers, we first transformed the metal concentrations to a normal distribution using the Box-Cox transformation (MASS package)^53,54^ and assessed outliers using Grubb’s test (outlier package)^55,56^. No significant outliers were detected using this method.

As there are no published standards for salivary metal concentrations considered clinically relevant, we categorized participants by tertiles of each metal. Alpha diversity for each tertile was assessed using the Shannon index and Chao1 with the Phyloseq package in R^57^. The diversity analysis was also conducted using a rarified dataset with 100 trials for comparison. Individuals in the first and second tertiles were combined to form a “low exposure” group. We used simple descriptive statistics (mean, median, standard deviation) to describe the distributions overall and by age. Because the distributions were highly skewed, we tested for differences in metal distribution by age and gender using the Wilcoxon Rank Sum test. Differences by personal income were tested using Kruskal–Wallis tests. For adults with missing income information and children we used the highest reported annual income from a family member in the same household when available.

For all metals where > 30% of participants had a value above the LOD, we next compared species abundance by tertile of metal exposure status using ALDEx2, an analysis of variance-like tool for compositional data^11^. For each species, ALDEx2 inferred absolute abundance given the observed abundance matrix using 1,000 Monte-Carlo simulations from a Dirichlet distribution to account for the fact that the observed abundance matrix is a single realization or sample from the oral bacterial communities of participants. The effect size for each species is the difference between groups relative to an estimate of within-group dispersion^58^. Statistical significance of the observed effect size between each metal and the relative abundance of each taxa was summarized as a p-value corrected for the false discovery rate using the Benjamini–Hochberg procedure. For simplicity, we summarize p-values associated with ALDEx2 tests and visualize statistically differences in species using association plots of species relative abundance. The significant associations were summarized in a table. ALDEx2 does not allow adjustment for covariates. Therefore, select taxa identified in the ALDEx2 analysis and additional taxa previously reported as associated with dental decay^59^ were further analyzed using linear mixed models. Separate models were fit using the lme4 R package^60^ to predict the relative abundance of the center-log ratio transformed relative abundance of each taxa by metal level, and included a random effect for family clustering.

We explored the relationship between salivary metal exposure (high, medium, and low) and presence of an untreated dental lesion using odds ratios from logistic regression models in SAS 9.4^52^. Due to the low percentage of detection with beryllium, thallium, uranium, and platinum, we compared the top 33% to the lower 66% for these metals. All models were adjusted for age as a continuous variable.

## Supplementary information

Supplementary file 1
