# Supplementary material for: Low levels of salivary metals, oral microbiome composition and dental decay
Source: Sci Rep. 2020 Sep 4;10:14640. doi: 10.1038/s41598-020-71495-9 (PMC7474081; doi:10.1038/s41598-020-71495-9)
Supplement: Supplementary file 1 [file 41598_2020_71495_MOESM1_ESM.docx]

**Supplemental Material**

**Low levels of salivary metals, oral microbiome composition and dental decay**

Elyse Davis^1^, Kelly Bakulski^2^, Jaclyn Goodrich^3^, Karen Peterson^3,4^, Mary Marazita^5^, Betsy Foxman^1^

^1^Center for Molecular and Clinical Epidemiology of Infectious Diseases, University of Michigan School of Public Health, 1415 Washington Heights, Ann Arbor, Michigan, 48109 USA.

^2^Department of Epidemiology, University of Michigan School of Public Health, 1415 Washington Heights, Ann Arbor, MI 48109

^3^Department of Environmental Health Sciences, University of Michigan School of Public Health, 1415 Washington Heights, Ann Arbor, MI 48109

^4^Department of Nutritional Sciences, University of Michigan School of Public Health, 1415 Washington Heights, Ann Arbor, MI 48109

^5^Center for Craniofacial and Dental Genetics, Department of Oral Biology, School of Dental Medicine; and Professor, Department of Human Genetics, Graduate School of Public Health; and Professor, Clinical and Translational Sciences Institute, School of Medicine, University of Pittsburgh, Pittsburgh, PA 15219.

**Table of Contents**

Exposures, risks, benefits, and limit of detection

for the essential minerals assessed in this study…………………………………..Table S1

Exposures, risks, benefits, and limit of detection

for the other metals assessed in this study…………………………………………Table S2

Exposures, risks, benefits, and limit of detection

for the metals with some known physiological benefits assessed in this study...Table S3

Chao1 and Shannon Diversity indices………………………………………………Table S4

Test statistics for differences in metal concentration by age, sex, and income...Table S5

Linear Mixed Model Estimates ………………………………………………………Table S6

*Suggested Lactobacilli species* ………………………………………..…………….Table S7

ALDEx2 results by age……………………………………… ……………………….Table S8

Odds ratios for dental decay adjusted for age and income ……………………….Table S9

Microbiome-wide associations of selected metals with salivary taxa……….…….Fig. S1

References

| **Table S1: Exposures, risks, benefits, and limit of detection for the essential minerals assessed in this study. Adapted from the Royal Society of Chemistry. (Last Accessed 4/29/2019)** | | | | | |
| --- | --- | --- | --- | --- | --- |
| **Essential Minerals** | | | | | |
| **Metal** | Environmental^61^ Exposure | Dietary Exposure | Risks/Benefits^61^ | Limit of Detection (ppb) | %>LOD |
| **Cobalt** | - Production of magnets, paint, porcelain, glass, pottery, and enamels | -Meat and dairy products^62^ | - forms part of the active site necessary for vitamin B12 | 0.1 | 100% |
| **Copper** | - Production of coins, and electrical equipment.  -Widely used in agriculture as a poison | - Shellfish, whole grains, beans, nuts, potatoes, dark leafy greens, and dried fruits^63^ | -used to help cells acquire energy from enzymes.  -too much copper is considered toxic | 10 | 99% |
| **Manganese** | -Production of safes, rifle barrels, and drink cans.  -Manganese oxide is found in fertilizers and ceramics. | -Nuts, bran, wholegrain cereals, tea, and parsley^61^ | - found in many types of enzymes  -necessary for vitamin B1 | 5 | 97% |
| **Molybdenum** | - Production of engines, drills, and saw blades | -Grains, legumes, nuts, and dairy products^64^ | -found in 50 different enzymes in plants and animals | 0.2 | 100% |
| **Zinc** | - Production of paints, rubber, cosmetics, plastics, inks, soaps, batteries, textiles, and electrical equipment | -Herring, beef, lamb, sunflower seeds, and cheese^61^ | -Forms the active site in over 20 metallo-enzymes | 30 | 100% |

| **Table S2: Exposures, risks, benefits, and limits of detection for heavy metals assessed in this study. Adapted from the Royal Society of Chemistry (last accessed 4/29/2019)** | | | | | |
| --- | --- | --- | --- | --- | --- |
| **Heavy Metals** | | | | | |
| **Metal** | Environmental^61^ Exposure | Dietary Exposure | Risks/Benefits^61^ | Limit of Detection (ppb) | %>LOD |
| **Antimony** | -Alloys used in the production of batteries, bullets  -Compounds are found in flame-retardant materials, paint, enamels, glass, and pottery | - Dietary exposure to antimony is low^65^  -May be present in drinking water^61^ | - Considered toxic | 0.1 | 100% |
| **Arsenic** | -Used in rat poisons, insecticides, glass, wood preservation  -Compounds are used in poultry farming to prevent disease and increase weight gain | -Seafood, rice, mushrooms and poultry^66^  - Drinking water | -Toxic in small doses  -Suspected carcinogen | 0.2 | 49% |
| **Barium** | -Used in drilling fluids, paint, and glassmaking  -Used in the clinical setting for x-raying gastrointestinal system | - Dietary exposure is not typically a concern for barium^67^  -Brazil nuts, seaweed, fish, and some plants^67^ | -Considered toxic | 0.5 | 100% |
| **Beryllium** | -Used in structural material and electrical work | - Raw carrots and field corn at very low levels^68^ | - Considered toxic and carcinogenic  -Inhalation can cause berylliosis in the lungs | 0.5 | 10% |
| **Cadmium** | - Used to make rechargeable batteries  -Tobacco smoke | -Leafy vegetables, potatoes, grains, soybeans, and sunflower seeds^69^ | -Considered toxic, carcinogenic, and teratogenic | 0.5 | 79% |
| **Cesium** | -Used to make drilling fluid | -Food grown in fields with radioactive cesium are at risk for  contamination^70^ | - Low hazard | 0.2 | 100% |
| **Lead** | - Used to produce car batteries, pigments, ammunition, and weights for lifting  -Paint in older homes | -Foods grown in soil with levels of lead are at risk for contamination^71^ | - Dangerous when accumulation in the body occurs  -Considered toxic, carcinogenic, and teratogenic | 0.1 | 100% |
| **Mercury** | -Dental amalgam fillings  -Released into environment from combustion of fossil fuels | -Fish | -Considered toxic even at low doses  -Particularly dangerous in the form of methylmercury | 0.1 | 70% |
| **Platinum** | -Used to produce jewelry, optic fibers, LCDs, pacemakers, dental fillings, and catalytic converters  -Used in the clinical setting in chemotherapy drugs | -May be found in potatoes^72^ | - No hazard known | 0.1 | 5% |
| **Thallium** | -Used to produce special glasses | -home-grown fruits and vegetables from contaminated soil^73^ | -Highly toxic, carcinogenic, and teratogenic  -Displaces potassium in the body to negatively impact the central nervous system | 0.5 | 7% |
| **Tin** | - Used to make cans  -Used to polish other metals | -canned food and juices^74^  -very low levels in fruits, vegetables, nuts, dairy products, and meat^74^ | -Non-toxic | 0.5 | 80% |
| **Uranium** | -Used to produce nuclear power | -potatoes, parsnips, and sweet potatoes, based on levels in soil^75^ | -No known biological role  -Toxic | 0.1 | 20% |

| **Table S3: Exposures, risks, benefits, and limits of detection for metals with some evidence of physiological function assessed in this study. Adapted from the Royal Society of Chemistry (last accessed 4/29/2019)** | | | | | |
| --- | --- | --- | --- | --- | --- |
| **Metals with Some Evidence of Physiological Function** | | | | | |
| **Metal** | Environmental Exposure^61^ | Dietary Exposure | Risks/Benefits^61^ | Limit of Detection (ppb) | %>LOD |
| **Chromium** | -Used to produce stainless steel, and plastic plating for bathroom fittings | -Fruits, nuts, vegetables, and meats^76^ | -Poisonous at high levels    -Aids in the use of glucose as a trace element. | 0.2 | 100% |
| **Nickel** | -Used to produce toasters and electric ovens, and batteries  -Used to plate other metals to prevent corrosion | -Chocolate, soybeans, nuts, and oatmeal^77^ | -Nickel allergies are relatively common.  -Biological role is unknown. | 2 | 100% |
| **Tungsten** | - Used in furnaces and electrodes  -A core component of metal, mining, and petroleum industries | -Dietary exposure is not a concern^78^ | - Some bacteria utilize tungsten to reduce carboxylic acids to aldehydes | 0.1 | 72% |
| **Vanadium** | - Used to produce steel alloys, ceramics, and glass | -High levels found in seafood^79^ | - Essential in very low amounts.  - It can be toxic at higher doses. | 0.1 | 80% |

| **Table S4: Shannon Diversity and Chao1 index for metals with more than 30% of participants above LOD. P.values presented are from Kruskall-wallis tests. Stimulated saliva samples were obtained from 61 individuals from the Center for Oral Health in Appalachia Study I.** | | | | | | | | | | |  | |  |  |
| --- | --- | --- | --- | --- | --- | --- | --- | --- | --- | --- | --- | --- | --- | --- |
|  | Shannon Diversity Index  Mean (SD) | | |  | Chao1 Richness  Mean (SD) | | | | | |  | |  |  |
| Metal | Low Exposure | Med  Exposure | High Exposure | p.value | Low  Exposure | | Med  Exposure | | High  Exposure | | | p.value |  |  |
| **Essential** |  |  |  |  |  | |  | |  | | |  |  |  |
| Cobalt | 4.90 (0.23) | 4.93 (0.18) | 4.90 (0.26) | 0.95 | 493 (2.61) | | 494 (2.05) | | 493 (4.07) | | | 0.84 |  |  |
| Copper | 4.89 (0.24) | 4.98 (0.20) | 4.98 (0.23) | 0.35 | 493 (2.46) | | 494 (4.14) | | 493 (2.02) | | | 0.69 |  |  |
| Manganese | 4.98 (0.21) | 4.88 (0.23) | 4.88 (0.23) | 0.21 | 493 (1.29) | | 493 (3.55) | | 494 (3.64) | | | 0.98 |  |  |
| Molybdenum | 4.98 (0.16) | 4.89 (0.24) | 4.86 (0.25) | 0.21 | 493 (1.27) | | 493 (1.53) | | 493 (4.90) | | | 0.69 |  |  |
| Zinc | 4.95 (0.23) | 4.95 (0.19) | 4.83 (0.23) | 0.15 | 492 (2.30) | | 493 (2.87) | | 494 (3.51) | | | 0.54 |  |  |
| **Heavy Metals** | |  |  |  |  | |  | |  | | |  |  |  |
| Antimony | 4.89 (0.21) | 4.94 (0.22) | 4.91 (0.24) | 0.69 | 493 (1.78) | | 493 (2.54) | | 494 (4.24) | | | 0.64 |  |  |
| Arsenic | 4.96 (0.24) | 4.91 (0.20) | 4.86 (0.22) | 0.17 | 494 (3.20) | | 493  (2.52) | | 493 (3.16) | | | 0.41 |  |  |
| Barium | 4.94 (0.21) | 4.92 (0.20) | 4.88 (0.26) | 0.73 | 493 (2.14) | | 492 (2.61) | | 495 (3.53) | | | 0.003 |  |  |
| Cadmium | 4.98 (0.18) | 4.84 (0.22) | 4.91 (0.25) | 0.08 | 493 (2.54) | | 493 (3.62) | | 494 (2.70) | | | 0.15 |  |  |
| Cesium | 4.98 (0.18) | 4.88 (0.27) | 4.88 (0.21) | 0.33 | 492 (2.23) | | 493 (1.77) | | 494 (4.22) | | | 0.12 |  |  |
| Lead | 4.98 (0.17) | 4.83 (0.26) | 4.93 (0.21) | 0.16 | 492 (2.47) | | 494 (3.96) | | 494 (2.18) | | | 0.19 |  |  |
| Mercury | 4.96 (0.19 | 4.91 (0.21) | 4.86 (0.26) | 0.46 | 493 (2.28) | | 493 (2.89) | | 494 (3.71) | | | 0.29 |  |  |
| Tin | 4.96 (0.20) | 4.95 (0.20) | 4.83 (0.25) | 0.19 | 493 (2.87) | | 493 (2.26) | | 494 (3.75) | | | 0.60 |  |  |
| **Some Evidence of Physiological Function** | | |  | |  |  | |  | |  | | |  |  |
| Chromium | 4.93 (0.17) | 4.94 (0.22) | 4.87 (0.27) | 0.61 | 493 (2.10) | | 494 (3.17) | | 492 (3.35) | | | 0.07 |  |  |
| Nickel | 4.97 (0.17) | 4.87 (0.25) | 4.89 (0.24) | 0.37 | 493 (2.67) | | 494 (1.66) | | 493 (4.19) | | | 0.31 |  |  |
| Tungsten | 4.95 (0.16) | 4.89 (0.26) | 4.89 (0.24) | 0.79 | 494 (2.33) | | 494 (3.30) | | 492 (3.01) | | | 0.11 |  |  |
| Vanadium | 4.98 (0.18) | 4.92 (0.19) | 4.83 (0.27) | 0.12 | 493 (2.69) | | 494 (2.07) | | 494 (3.99) | | | 0.69 |  |  |

| **Table S5: Results of tests between metal concentration and age, sex, and income. Cesium levels were significantly associated with age and copper with income (shown in bold). Stimulated saliva samples were obtained from 61 individuals from the Center for Oral Health in Appalachia Study I.** | | | | | | | | |
| --- | --- | --- | --- | --- | --- | --- | --- | --- |
|  | Age | |  | Sex | |  | Income | |
| Metal | Test Statistic* | p.value |  | Test Statistic* | p.value |  | Test Statistic** | p.value |
| **Essential** |  |  |  |  |  |  |  |  |
| Cobalt | 283 | 0.62 |  | 476 | 0.64 |  | 0.14 | 0.93 |
| Copper | 216 | 0.09 |  | 499 | 0.42 |  | 6.69 | **0.04** |
| Manganese | 339 | 0.65 |  | 531 | 0.20 |  | 0.58 | 0.75 |
| Molybdenum | 308 | 0.95 |  | 406 | 0.58 |  | 1.26 | 0.53 |
| Zinc | 313 | 0.99 |  | 510 | 0.34 |  | 0.95 | 0.62 |
| **Heavy Metals** | |  |  |  |  |  |  |  |
| Antimony | 357 | 0.44 |  | 467 | 0.74 |  | 5.19 | 0.07 |
| Arsenic | 316 | 0.95 |  | 391 | 0.41 |  | 1.16 | 0.56 |
| Barium | 374 | 0.28 |  | 531 | 0.20 |  | 4.28 | 0.12 |
| Cadmium | 299 | 0.82 |  | 539 | 0.16 |  | 1.61 | 0.45 |
| Cesium | 448 | **0.02** |  | 515 | 0.30 |  | 1.16 | 0.56 |
| Lead | 271 | 0.48 |  | 544 | 0.14 |  | 3.57 | 0.17 |
| Mercury | 385 | 0.20 |  | 506 | 0.36 |  | 0.005 | 0.92 |
| Tin | 311 | 0.99 |  | 478 | 0.62 |  | 0.32 | 0.85 |
| Beryllium | 255 | 0.05 |  | 465 | 0.56 |  | 0.25 | 0.88 |
| Platinum | 300 | 0.59 |  | 469 | 0.33 |  | 3.15 | 0.19 |
| Thallium | 275 | 0.13 |  | 459 | 0.62 |  | 0.42 | 0.81 |
| Uranium | 294 | 0.66 |  | 489 | 0.34 |  | 0.44 | 0.80 |
| **Some Evidence of Physiological Function** | | |  |  | |  |  |  |
| Chromium | 219 | 0.10 |  | 387 | 0.41 |  | 5.18 | 0.07 |
| Nickel | 244 | 0.24 |  | 420 | 0.73 |  | 2.42 | 0.30 |
| Tungsten | 206 | 0.06 |  | 410 | 0.62 |  | 3.31 | 0.19 |
| Vanadium | 328 | 0.78 |  | 412 | 0.64 |  | 0.21 | 0.90 |
| *Wilcoxon rank-sum test  **Kruskal-Wallis test with 2 degrees of freedom. Income groups: <$10,000; 10,000-35,000; 35,000+ | | | | | | | | |

| **Table S6. Estimates from linear mixed models showing the associations between centered-log ratio transformed species counts of taxa significant in the ALDEx2 analysis (noted with *), selected cariogenic taxa, and antimony exposure. Metal levels were modeled as high and low exposure, with low as the reference group. All models include a random effect for family clustering. Direction of ALDEx2 effect sizes are included for comparison.** | | |
| --- | --- | --- |
| **Species** | **Linear Mixed Model Estimate** | **ALDEx2 Results** |
| *Escherichia coli* | 0.9714* | Increase** |
| *Lactobacillus_sp_11809* | 1.0540* | Increase** |
| *Lactobacillus_sp_11808* | 1.0342* | Increase** |
| *Lactobacillus_sp_11800* | 0.9956* | Increase** |
| *Lactobacillus_sp_11798* | 1.0566 * | Increase** |
| *Streptococcus_gordonii* | 0.1291 | Increase |
| *Streptococcus_vestibularis_salivarius* | -0.2194 | Decrease |
| *Streptococcus_parasanguinis* | -0.15133 | Decrease |
| *Veillonella_atypica_dispar_parvula* | -0.12783 | Decrease |
| * p.value < 0.05  ** Benjamini-Hochberg p.value < 0.10 | | |

| **Table S7. Top suggested species level identification from NCBI Basic Local Alignment Search Tool for unspecified Lactobacilli species significantly associated with antimony exposure levels.** | | | | | | |
| --- | --- | --- | --- | --- | --- | --- |
| *Lactobacillus sp* Number | Description | Max Score | Query Cover | E value | Percent identity | Accession Number |
| 11798 | Lactobacillus acidipiscis strain 3706 16S ribosomal RNA gene, partial sequence | 171 | 95% | 6.00E-39 | 100 | [MT538565.1](https://www.ncbi.nlm.nih.gov/nucleotide/MT538565.1?report=genbank&log$=nucltop&blast_rank=1&RID=HBCJHV6J014) |
| 11798 | Lactobacillus acidipiscis strain 3655 16S ribosomal RNA gene, partial sequence | 171 | 95% | 6.00E-39 | 100 | [MT538521.1](https://www.ncbi.nlm.nih.gov/nucleotide/MT538521.1?report=genbank&log$=nucltop&blast_rank=2&RID=HBCJHV6J014) |
| 11798 | Lactobacillus acidipiscis strain 3506 16S ribosomal RNA gene, partial sequence | 171 | 95% | 6.00E-39 | 100 | [MT538406.1](https://www.ncbi.nlm.nih.gov/nucleotide/MT538406.1?report=genbank&log$=nucltop&blast_rank=3&RID=HBCJHV6J014) |
| 11798 | Lactobacillus acidipiscis strain 3454 16S ribosomal RNA gene, partial sequence | 171 | 95% | 6.00E-39 | 100 | [MT538362.1](https://www.ncbi.nlm.nih.gov/nucleotide/MT538362.1?report=genbank&log$=nucltop&blast_rank=4&RID=HBCJHV6J014) |
| 11798 | Lactobacillus acidipiscis strain 7258 16S ribosomal RNA gene, partial sequence | 171 | 95% | 6.00E-39 | 100 | [MT516054.1](https://www.ncbi.nlm.nih.gov/nucleotide/MT516054.1?report=genbank&log$=nucltop&blast_rank=5&RID=HBCJHV6J014) |
| 11798 | Lactobacillus acidipiscis strain 7036 16S ribosomal RNA gene, partial sequence | 171 | 95% | 6.00E-39 | 100 | [MT464082.1](https://www.ncbi.nlm.nih.gov/nucleotide/MT464082.1?report=genbank&log$=nucltop&blast_rank=6&RID=HBCJHV6J014) |
| 11798 | Lactobacillus pobuzihii JCM 18084 gene for 16S rRNA, partial sequence | 171 | 95% | 6.00E-39 | 100 | [LC521982.1](https://www.ncbi.nlm.nih.gov/nucleotide/LC521982.1?report=genbank&log$=nucltop&blast_rank=7&RID=HBCJHV6J014) |
| 11798 | Lactobacillus salitolerans gene for 16S ribosomal RNA, partial sequence | 171 | 95% | 6.00E-39 | 100 | [LC127508.1](https://www.ncbi.nlm.nih.gov/nucleotide/LC127508.1?report=genbank&log$=nucltop&blast_rank=8&RID=HBCJHV6J014) |
| 11798 | Lactobacillus acidipiscis strain JC1132 16S ribosomal RNA gene, partial sequence | 171 | 95% | 6.00E-39 | 100 | [MH819758.1](https://www.ncbi.nlm.nih.gov/nucleotide/MH819758.1?report=genbank&log$=nucltop&blast_rank=9&RID=HBCJHV6J014) |
| 11798 | Lactobacillus acidipiscis strain HBUAS52446 16S ribosomal RNA gene, partial sequence | 171 | 95% | 6.00E-39 | 100 | [MK396552.1](https://www.ncbi.nlm.nih.gov/nucleotide/MK396552.1?report=genbank&log$=nucltop&blast_rank=10&RID=HBCJHV6J014) |
| 11798 | Lactobacillus acidipiscis strain Z47A 16S ribosomal RNA gene, partial sequence | 171 | 95% | 6.00E-39 | 100 | [MG050119.1](https://www.ncbi.nlm.nih.gov/nucleotide/MG050119.1?report=genbank&log$=nucltop&blast_rank=11&RID=HBCJHV6J014) |
| 11798 | Lactobacillus sp. strain FE-1 16S ribosomal RNA gene, partial sequence | 171 | 95% | 6.00E-39 | 100 | [MG042082.1](https://www.ncbi.nlm.nih.gov/nucleotide/MG042082.1?report=genbank&log$=nucltop&blast_rank=12&RID=HBCJHV6J014) |
| 11798 | Lactobacillus acidipiscis strain VP-3.5 16S ribosomal RNA gene, partial sequence | 171 | 95% | 6.00E-39 | 100 | [MF191703.1](https://www.ncbi.nlm.nih.gov/nucleotide/MF191703.1?report=genbank&log$=nucltop&blast_rank=13&RID=HBCJHV6J014) |
| 11798 | Lactobacillus acidipiscis strain VP-2.5 16S ribosomal RNA gene, partial sequence | 171 | 95% | 6.00E-39 | 100 | [MF191699.1](https://www.ncbi.nlm.nih.gov/nucleotide/MF191699.1?report=genbank&log$=nucltop&blast_rank=14&RID=HBCJHV6J014) |
| 11798 | Uncultured Lactobacillus sp. clone xw2-70 16S ribosomal RNA gene, partial sequence | 171 | 95% | 6.00E-39 | 100 | [KU961712.1](https://www.ncbi.nlm.nih.gov/nucleotide/KU961712.1?report=genbank&log$=nucltop&blast_rank=15&RID=HBCJHV6J014) |
| 11798 | Lactobacillus sp. strain 27-1 16S ribosomal RNA gene, partial sequence | 171 | 95% | 6.00E-39 | 100 | [KX499358.1](https://www.ncbi.nlm.nih.gov/nucleotide/KX499358.1?report=genbank&log$=nucltop&blast_rank=16&RID=HBCJHV6J014) |
| 11798 | Lactobacillus acidipiscis strain PR09 16S ribosomal RNA gene, partial sequence | 171 | 95% | 6.00E-39 | 100 | [KX139191.1](https://www.ncbi.nlm.nih.gov/nucleotide/KX139191.1?report=genbank&log$=nucltop&blast_rank=17&RID=HBCJHV6J014) |
| 11798 | Uncultured bacterium clone OTU_2465 16S ribosomal RNA gene, partial sequence | 171 | 95% | 6.00E-39 | 100 | [KU650871.1](https://www.ncbi.nlm.nih.gov/nucleotide/KU650871.1?report=genbank&log$=nucltop&blast_rank=18&RID=HBCJHV6J014) |
| 11798 | Lactobacillus sp. K30A 16S ribosomal RNA gene, partial sequence | 171 | 95% | 6.00E-39 | 100 | [KU714846.1](https://www.ncbi.nlm.nih.gov/nucleotide/KU714846.1?report=genbank&log$=nucltop&blast_rank=19&RID=HBCJHV6J014) |
| 11798 | Lactobacillus sp. K29C 16S ribosomal RNA gene, partial sequence | 171 | 95% | 6.00E-39 | 100 | [KU714845.1](https://www.ncbi.nlm.nih.gov/nucleotide/KU714845.1?report=genbank&log$=nucltop&blast_rank=20&RID=HBCJHV6J014) |
| 11798 | Lactobacillus acidipiscis gene for 16S ribosomal RNA, partial sequence, strain: SR7-1 | 171 | 95% | 6.00E-39 | 100 | [LC127171.1](https://www.ncbi.nlm.nih.gov/nucleotide/LC127171.1?report=genbank&log$=nucltop&blast_rank=21&RID=HBCJHV6J014) |
| 11798 | Lactobacillus acidipiscis partial 16S rRNA gene, strain MT19 | 171 | 95% | 6.00E-39 | 100 | [LN898271.1](https://www.ncbi.nlm.nih.gov/nucleotide/LN898271.1?report=genbank&log$=nucltop&blast_rank=22&RID=HBCJHV6J014) |
| 11798 | Lactobacillus sp. B4(2014) 16S ribosomal RNA gene, partial sequence | 171 | 95% | 6.00E-39 | 100 | [KM259929.1](https://www.ncbi.nlm.nih.gov/nucleotide/KM259929.1?report=genbank&log$=nucltop&blast_rank=23&RID=HBCJHV6J014) |
| 11798 | Uncultured prokaryote clone OTU100 16S ribosomal RNA gene, partial sequence | 171 | 95% | 6.00E-39 | 100 | [KF358873.1](https://www.ncbi.nlm.nih.gov/nucleotide/KF358873.1?report=genbank&log$=nucltop&blast_rank=24&RID=HBCJHV6J014) |
| 11798 | Uncultured Lactobacillus sp. gene for 16S ribosomal RNA, partial sequence, clone: 4X13 | 171 | 95% | 6.00E-39 | 100 | [LC002953.1](https://www.ncbi.nlm.nih.gov/nucleotide/LC002953.1?report=genbank&log$=nucltop&blast_rank=25&RID=HBCJHV6J014) |
| 11798 | Lactobacillus acidipiscis isolate ITA44 16S ribosomal RNA gene, partial sequence | 171 | 95% | 6.00E-39 | 100 | [KF297816.1](https://www.ncbi.nlm.nih.gov/nucleotide/KF297816.1?report=genbank&log$=nucltop&blast_rank=26&RID=HBCJHV6J014) |
| 11798 | Pediococcus sp. F3S1 16S ribosomal RNA gene, partial sequence | 171 | 95% | 6.00E-39 | 100 | [KF245543.1](https://www.ncbi.nlm.nih.gov/nucleotide/KF245543.1?report=genbank&log$=nucltop&blast_rank=27&RID=HBCJHV6J014) |
| 11798 | Uncultured Lactobacillus sp. clone 3c8 16S ribosomal RNA gene, partial sequence | 171 | 95% | 6.00E-39 | 100 | [KC755062.1](https://www.ncbi.nlm.nih.gov/nucleotide/KC755062.1?report=genbank&log$=nucltop&blast_rank=28&RID=HBCJHV6J014) |
| 11798 | Uncultured bacterium clone OTU93 16S ribosomal RNA gene, partial sequence | 171 | 95% | 6.00E-39 | 100 | [KC120652.1](https://www.ncbi.nlm.nih.gov/nucleotide/KC120652.1?report=genbank&log$=nucltop&blast_rank=29&RID=HBCJHV6J014) |
| 11798 | Lactobacillus acidipiscis gene for 16S rRNA, partial sequence, strain: Ni1465 | 171 | 95% | 6.00E-39 | 100 | [AB598991.1](https://www.ncbi.nlm.nih.gov/nucleotide/AB598991.1?report=genbank&log$=nucltop&blast_rank=30&RID=HBCJHV6J014) |
| 11798 | Lactobacillus acidipiscis gene for 16S rRNA, partial sequence, strain: Ni1462 | 171 | 95% | 6.00E-39 | 100 | [AB598989.1](https://www.ncbi.nlm.nih.gov/nucleotide/AB598989.1?report=genbank&log$=nucltop&blast_rank=31&RID=HBCJHV6J014) |
| 11798 | Lactobacillus acidipiscis gene for 16S rRNA, partial sequence, strain: Ni958 | 171 | 95% | 6.00E-39 | 100 | [AB598946.1](https://www.ncbi.nlm.nih.gov/nucleotide/AB598946.1?report=genbank&log$=nucltop&blast_rank=32&RID=HBCJHV6J014) |
| 11798 | Uncultured Lactobacillus sp. clone SCTB031 16S ribosomal RNA gene, partial sequence | 171 | 95% | 6.00E-39 | 100 | [JN650258.1](https://www.ncbi.nlm.nih.gov/nucleotide/JN650258.1?report=genbank&log$=nucltop&blast_rank=33&RID=HBCJHV6J014) |
| 11798 | Lactobacillus pobuzihii strain LZLJ22-3 16S ribosomal RNA gene, partial sequence | 171 | 95% | 6.00E-39 | 100 | [JQ043379.1](https://www.ncbi.nlm.nih.gov/nucleotide/JQ043379.1?report=genbank&log$=nucltop&blast_rank=34&RID=HBCJHV6J014) |
| 11798 | Lactobacillus similis strain LZLJ22-1 16S ribosomal RNA gene, partial sequence | 171 | 95% | 6.00E-39 | 100 | [JQ043378.1](https://www.ncbi.nlm.nih.gov/nucleotide/JQ043378.1?report=genbank&log$=nucltop&blast_rank=35&RID=HBCJHV6J014) |
| 11798 | Lactobacillus acidipiscis strain LZLJ19-3 16S ribosomal RNA gene, partial sequence | 171 | 95% | 6.00E-39 | 100 | [JQ043377.1](https://www.ncbi.nlm.nih.gov/nucleotide/JQ043377.1?report=genbank&log$=nucltop&blast_rank=36&RID=HBCJHV6J014) |
| 11798 | Lactobacillus acidipiscis strain LZLJ18-3 16S ribosomal RNA gene, partial sequence | 171 | 95% | 6.00E-39 | 100 | [JQ043376.1](https://www.ncbi.nlm.nih.gov/nucleotide/JQ043376.1?report=genbank&log$=nucltop&blast_rank=37&RID=HBCJHV6J014) |
| 11798 | Lactobacillus acidipiscis strain LZLJ12-3 16S ribosomal RNA gene, partial sequence | 171 | 95% | 6.00E-39 | 100 | [JQ043374.1](https://www.ncbi.nlm.nih.gov/nucleotide/JQ043374.1?report=genbank&log$=nucltop&blast_rank=38&RID=HBCJHV6J014) |
| 11798 | Lactobacillus acidipiscis strain LZLJ10-1 16S ribosomal RNA gene, partial sequence | 171 | 95% | 6.00E-39 | 100 | [JQ043372.1](https://www.ncbi.nlm.nih.gov/nucleotide/JQ043372.1?report=genbank&log$=nucltop&blast_rank=39&RID=HBCJHV6J014) |
| 11798 | Lactobacillus acidipiscis strain LZLJ8-3 16S ribosomal RNA gene, partial sequence | 171 | 95% | 6.00E-39 | 100 | [JQ043371.1](https://www.ncbi.nlm.nih.gov/nucleotide/JQ043371.1?report=genbank&log$=nucltop&blast_rank=40&RID=HBCJHV6J014) |
| 11798 | Lactobacillus acidipiscis strain LZLJ8-1 16S ribosomal RNA gene, partial sequence | 171 | 95% | 6.00E-39 | 100 | [JQ043370.1](https://www.ncbi.nlm.nih.gov/nucleotide/JQ043370.1?report=genbank&log$=nucltop&blast_rank=41&RID=HBCJHV6J014) |
| 11798 | Lactobacillus acidipiscis strain LZLJ4-3 16S ribosomal RNA gene, partial sequence | 171 | 95% | 6.00E-39 | 100 | [JQ043369.1](https://www.ncbi.nlm.nih.gov/nucleotide/JQ043369.1?report=genbank&log$=nucltop&blast_rank=42&RID=HBCJHV6J014) |
| 11798 | Lactobacillus acidipiscis strain LZLJ4-1 16S ribosomal RNA gene, partial sequence | 171 | 95% | 6.00E-39 | 100 | [JQ043368.1](https://www.ncbi.nlm.nih.gov/nucleotide/JQ043368.1?report=genbank&log$=nucltop&blast_rank=43&RID=HBCJHV6J014) |
| 11798 | Lactobacillus acidipiscis strain LZLJ2-3 16S ribosomal RNA gene, partial sequence | 171 | 95% | 6.00E-39 | 100 | [JQ043367.1](https://www.ncbi.nlm.nih.gov/nucleotide/JQ043367.1?report=genbank&log$=nucltop&blast_rank=44&RID=HBCJHV6J014) |
| 11798 | Lactobacillus sp. TRF8 16S ribosomal RNA gene, partial sequence | 171 | 95% | 6.00E-39 | 100 | [JN660076.1](https://www.ncbi.nlm.nih.gov/nucleotide/JN660076.1?report=genbank&log$=nucltop&blast_rank=45&RID=HBCJHV6J014) |
| 11798 | Lactobacillus sp. TRF5 16S ribosomal RNA gene, partial sequence | 171 | 95% | 6.00E-39 | 100 | [JN660074.1](https://www.ncbi.nlm.nih.gov/nucleotide/JN660074.1?report=genbank&log$=nucltop&blast_rank=46&RID=HBCJHV6J014) |
| 11798 | Lactobacillus sp. TRF7 16S ribosomal RNA gene, partial sequence | 171 | 95% | 6.00E-39 | 100 | [JN660059.1](https://www.ncbi.nlm.nih.gov/nucleotide/JN660059.1?report=genbank&log$=nucltop&blast_rank=47&RID=HBCJHV6J014) |
| 11798 | Lactobacillus sp. TTp14 16S ribosomal RNA gene, partial sequence | 171 | 95% | 6.00E-39 | 100 | [HQ141624.1](https://www.ncbi.nlm.nih.gov/nucleotide/HQ141624.1?report=genbank&log$=nucltop&blast_rank=48&RID=HBCJHV6J014) |
| 11798 | Lactobacillus sp. TTp13 16S ribosomal RNA gene, partial sequence | 171 | 95% | 6.00E-39 | 100 | [HQ141623.1](https://www.ncbi.nlm.nih.gov/nucleotide/HQ141623.1?report=genbank&log$=nucltop&blast_rank=49&RID=HBCJHV6J014) |
| 11798 | Lactobacillus sp. TTp12 16S ribosomal RNA gene, partial sequence | 171 | 95% | 6.00E-39 | 100 | [HQ141622.1](https://www.ncbi.nlm.nih.gov/nucleotide/HQ141622.1?report=genbank&log$=nucltop&blast_rank=50&RID=HBCJHV6J014) |
| 11798 | Lactobacillus sp. TTp6 16S ribosomal RNA gene, partial sequence | 171 | 95% | 6.00E-39 | 100 | [HQ141621.1](https://www.ncbi.nlm.nih.gov/nucleotide/HQ141621.1?report=genbank&log$=nucltop&blast_rank=51&RID=HBCJHV6J014) |
| 11798 | Lactobacillus sp. TTp4 16S ribosomal RNA gene, partial sequence | 171 | 95% | 6.00E-39 | 100 | [HQ141620.1](https://www.ncbi.nlm.nih.gov/nucleotide/HQ141620.1?report=genbank&log$=nucltop&blast_rank=52&RID=HBCJHV6J014) |
| 11798 | Lactobacillus sp. Tporo2 16S ribosomal RNA gene, partial sequence | 171 | 95% | 6.00E-39 | 100 | [HM534775.1](https://www.ncbi.nlm.nih.gov/nucleotide/HM534775.1?report=genbank&log$=nucltop&blast_rank=53&RID=HBCJHV6J014) |
| 11798 | Lactobacillus sp. MbBipro17 16S ribosomal RNA gene, partial sequence | 171 | 95% | 6.00E-39 | 100 | [HM534774.1](https://www.ncbi.nlm.nih.gov/nucleotide/HM534774.1?report=genbank&log$=nucltop&blast_rank=54&RID=HBCJHV6J014) |
| 11798 | Lactobacillus sp. Thmro2 16S ribosomal RNA gene, partial sequence | 171 | 95% | 6.00E-39 | 100 | [HM534773.1](https://www.ncbi.nlm.nih.gov/nucleotide/HM534773.1?report=genbank&log$=nucltop&blast_rank=55&RID=HBCJHV6J014) |
| 11798 | Lactobacillus acidipiscis strain 5-1 16S ribosomal RNA gene, partial sequence | 171 | 95% | 6.00E-39 | 100 | [HQ384296.1](https://www.ncbi.nlm.nih.gov/nucleotide/HQ384296.1?report=genbank&log$=nucltop&blast_rank=56&RID=HBCJHV6J014) |
| 11798 | Lactobacillus pobuzihii gene for 16S rRNA, partial sequence, strain: NBRC 103227 | 171 | 95% | 6.00E-39 | 100 | [AB326366.1](https://www.ncbi.nlm.nih.gov/nucleotide/AB326366.1?report=genbank&log$=nucltop&blast_rank=57&RID=HBCJHV6J014) |
| 11798 | Lactobacillus pobuzihii gene for 16S rRNA, partial sequence, strain: NBRC 103228 | 171 | 95% | 6.00E-39 | 100 | [AB326367.1](https://www.ncbi.nlm.nih.gov/nucleotide/AB326367.1?report=genbank&log$=nucltop&blast_rank=58&RID=HBCJHV6J014) |
| 11798 | Lactobacillus pobuzihii gene for 16S rRNA, partial sequence, strain: NBRC 103226 | 171 | 95% | 6.00E-39 | 100 | [AB326365.1](https://www.ncbi.nlm.nih.gov/nucleotide/AB326365.1?report=genbank&log$=nucltop&blast_rank=59&RID=HBCJHV6J014) |
| 11798 | Lactobacillus pobuzihii strain E100301 16S ribosomal RNA, partial sequence | 171 | 95% | 6.00E-39 | 100 | [NR_112694.1](https://www.ncbi.nlm.nih.gov/nucleotide/NR_112694.1?report=genbank&log$=nucltop&blast_rank=60&RID=HBCJHV6J014) |
| 11798 | Uncultured bacterium clone l_A23 16S ribosomal RNA gene, partial sequence | 171 | 95% | 6.00E-39 | 100 | [EU448891.1](https://www.ncbi.nlm.nih.gov/nucleotide/EU448891.1?report=genbank&log$=nucltop&blast_rank=61&RID=HBCJHV6J014) |
| 11798 | Lactobacillus acidipiscis gene for 16S rRNA, partial sequence, strain: NBRC 102164 | 171 | 95% | 6.00E-39 | 100 | [AB326357.1](https://www.ncbi.nlm.nih.gov/nucleotide/AB326357.1?report=genbank&log$=nucltop&blast_rank=62&RID=HBCJHV6J014) |
| 11798 | Uncultured compost bacterium clone 0B21 16S ribosomal RNA gene, partial sequence | 171 | 95% | 6.00E-39 | 100 | [DQ345474.1](https://www.ncbi.nlm.nih.gov/nucleotide/DQ345474.1?report=genbank&log$=nucltop&blast_rank=63&RID=HBCJHV6J014) |
| 11798 | Lactobacillus sp. YE06 16S ribosomal RNA gene, partial sequence | 171 | 95% | 6.00E-39 | 100 | [AF316585.1](https://www.ncbi.nlm.nih.gov/nucleotide/AF316585.1?report=genbank&log$=nucltop&blast_rank=64&RID=HBCJHV6J014) |
| 11798 | Lactobacillus sp. 121B 16S ribosomal RNA gene, partial sequence | 171 | 95% | 6.00E-39 | 100 | [AF305930.1](https://www.ncbi.nlm.nih.gov/nucleotide/AF305930.1?report=genbank&log$=nucltop&blast_rank=65&RID=HBCJHV6J014) |
| 11798 | Lactobacillus sp. LMK3 16S rRNA gene, strain LMK3 | 171 | 95% | 6.00E-39 | 100 | [AJ251560.1](https://www.ncbi.nlm.nih.gov/nucleotide/AJ251560.1?report=genbank&log$=nucltop&blast_rank=66&RID=HBCJHV6J014) |
| 11798 | Lactobacillus sp. FS1111 gene for 16S rRNA, partial sequence | 171 | 95% | 6.00E-39 | 100 | [AB023837.1](https://www.ncbi.nlm.nih.gov/nucleotide/AB023837.1?report=genbank&log$=nucltop&blast_rank=67&RID=HBCJHV6J014) |
| 11798 | Lactobacillus acidipiscis strain FS60-1 16S ribosomal RNA, partial sequence | 171 | 95% | 6.00E-39 | 100 | [NR_024718.1](https://www.ncbi.nlm.nih.gov/nucleotide/NR_024718.1?report=genbank&log$=nucltop&blast_rank=68&RID=HBCJHV6J014) |
| 11800 | Lactobacillus acidipiscis strain BP-1.2 16S ribosomal RNA gene, partial sequence | 171 | 95% | 6.00E-39 | 100 | [MF191678.1](https://www.ncbi.nlm.nih.gov/nucleotide/MF191678.1?report=genbank&log$=nucltop&blast_rank=1&RID=HBCM0J3X014) |
| 11800 | Uncultured Lactobacillus sp. clone xw3-167 16S ribosomal RNA gene, partial sequence | 171 | 95% | 6.00E-39 | 100 | [KU961720.1](https://www.ncbi.nlm.nih.gov/nucleotide/KU961720.1?report=genbank&log$=nucltop&blast_rank=2&RID=HBCM0J3X014) |
| 11800 | Lactobacillus acidipiscis strain ACA-DC 1533 genome assembly, chromosome: I | 171 | 95% | 6.00E-39 | 100 | [LT630287.1](https://www.ncbi.nlm.nih.gov/nucleotide/LT630287.1?report=genbank&log$=nucltop&blast_rank=3&RID=HBCM0J3X014) |
| 11800 | Uncultured Lactobacillus sp. clone SCTB037 16S ribosomal RNA gene, partial sequence | 171 | 95% | 6.00E-39 | 100 | [JN650260.1](https://www.ncbi.nlm.nih.gov/nucleotide/JN650260.1?report=genbank&log$=nucltop&blast_rank=4&RID=HBCM0J3X014) |
| 11800 | Lactobacillus sp. NBRC 107294 gene for 16S rRNA, partial sequence | 171 | 95% | 6.00E-39 | 100 | [AB682589.1](https://www.ncbi.nlm.nih.gov/nucleotide/AB682589.1?report=genbank&log$=nucltop&blast_rank=5&RID=HBCM0J3X014) |
| 11800 | Lactobacillus sp. NBRC 107255 gene for 16S rRNA, partial sequence | 171 | 95% | 6.00E-39 | 100 | [AB682553.1](https://www.ncbi.nlm.nih.gov/nucleotide/AB682553.1?report=genbank&log$=nucltop&blast_rank=6&RID=HBCM0J3X014) |
| 11800 | Uncultured compost bacterium clone 0B31 16S ribosomal RNA gene, partial sequence | 171 | 95% | 6.00E-39 | 100 | [DQ345484.1](https://www.ncbi.nlm.nih.gov/nucleotide/DQ345484.1?report=genbank&log$=nucltop&blast_rank=7&RID=HBCM0J3X014) |
| 11808 | Lactobacillus acidipiscis strain 3706 16S ribosomal RNA gene, partial sequence | 165 | 95% | 3.00E-37 | 98.91 | [MT538565.1](https://www.ncbi.nlm.nih.gov/nucleotide/MT538565.1?report=genbank&log$=nucltop&blast_rank=1&RID=HBCPDMXD016) |
| 11808 | Lactobacillus acidipiscis strain 3655 16S ribosomal RNA gene, partial sequence | 165 | 95% | 3.00E-37 | 98.91 | [MT538521.1](https://www.ncbi.nlm.nih.gov/nucleotide/MT538521.1?report=genbank&log$=nucltop&blast_rank=2&RID=HBCPDMXD016) |
| 11808 | Lactobacillus acidipiscis strain 3506 16S ribosomal RNA gene, partial sequence | 165 | 95% | 3.00E-37 | 98.91 | [MT538406.1](https://www.ncbi.nlm.nih.gov/nucleotide/MT538406.1?report=genbank&log$=nucltop&blast_rank=3&RID=HBCPDMXD016) |
| 11808 | Lactobacillus acidipiscis strain 3454 16S ribosomal RNA gene, partial sequence | 165 | 95% | 3.00E-37 | 98.91 | [MT538362.1](https://www.ncbi.nlm.nih.gov/nucleotide/MT538362.1?report=genbank&log$=nucltop&blast_rank=4&RID=HBCPDMXD016) |
| 11808 | Lactobacillus acidipiscis strain 7258 16S ribosomal RNA gene, partial sequence | 165 | 95% | 3.00E-37 | 98.91 | [MT516054.1](https://www.ncbi.nlm.nih.gov/nucleotide/MT516054.1?report=genbank&log$=nucltop&blast_rank=5&RID=HBCPDMXD016) |
| 11808 | Lactobacillus acidipiscis strain 7036 16S ribosomal RNA gene, partial sequence | 165 | 95% | 3.00E-37 | 98.91 | [MT464082.1](https://www.ncbi.nlm.nih.gov/nucleotide/MT464082.1?report=genbank&log$=nucltop&blast_rank=6&RID=HBCPDMXD016) |
| 11808 | Lactobacillus pobuzihii JCM 18084 gene for 16S rRNA, partial sequence | 165 | 95% | 3.00E-37 | 98.91 | [LC521982.1](https://www.ncbi.nlm.nih.gov/nucleotide/LC521982.1?report=genbank&log$=nucltop&blast_rank=7&RID=HBCPDMXD016) |
| 11808 | Lactobacillus salitolerans gene for 16S ribosomal RNA, partial sequence | 165 | 95% | 3.00E-37 | 98.91 | [LC127508.1](https://www.ncbi.nlm.nih.gov/nucleotide/LC127508.1?report=genbank&log$=nucltop&blast_rank=8&RID=HBCPDMXD016) |
| 11808 | Lactobacillus acidipiscis strain JC1132 16S ribosomal RNA gene, partial sequence | 165 | 95% | 3.00E-37 | 98.91 | [MH819758.1](https://www.ncbi.nlm.nih.gov/nucleotide/MH819758.1?report=genbank&log$=nucltop&blast_rank=9&RID=HBCPDMXD016) |
| 11808 | Lactobacillus acidipiscis strain HBUAS52446 16S ribosomal RNA gene, partial sequence | 165 | 95% | 3.00E-37 | 98.91 | [MK396552.1](https://www.ncbi.nlm.nih.gov/nucleotide/MK396552.1?report=genbank&log$=nucltop&blast_rank=10&RID=HBCPDMXD016) |
| 11808 | Lactobacillus acidipiscis strain Z47A 16S ribosomal RNA gene, partial sequence | 165 | 95% | 3.00E-37 | 98.91 | [MG050119.1](https://www.ncbi.nlm.nih.gov/nucleotide/MG050119.1?report=genbank&log$=nucltop&blast_rank=11&RID=HBCPDMXD016) |
| 11808 | Lactobacillus sp. strain FE-1 16S ribosomal RNA gene, partial sequence | 165 | 95% | 3.00E-37 | 98.91 | [MG042082.1](https://www.ncbi.nlm.nih.gov/nucleotide/MG042082.1?report=genbank&log$=nucltop&blast_rank=12&RID=HBCPDMXD016) |
| 11808 | Lactobacillus acidipiscis strain VP-3.5 16S ribosomal RNA gene, partial sequence | 165 | 95% | 3.00E-37 | 98.91 | [MF191703.1](https://www.ncbi.nlm.nih.gov/nucleotide/MF191703.1?report=genbank&log$=nucltop&blast_rank=13&RID=HBCPDMXD016) |
| 11808 | Lactobacillus acidipiscis strain VP-2.5 16S ribosomal RNA gene, partial sequence | 165 | 95% | 3.00E-37 | 98.91 | [MF191699.1](https://www.ncbi.nlm.nih.gov/nucleotide/MF191699.1?report=genbank&log$=nucltop&blast_rank=14&RID=HBCPDMXD016) |
| 11808 | Uncultured Lactobacillus sp. clone xw2-70 16S ribosomal RNA gene, partial sequence | 165 | 95% | 3.00E-37 | 98.91 | [KU961712.1](https://www.ncbi.nlm.nih.gov/nucleotide/KU961712.1?report=genbank&log$=nucltop&blast_rank=15&RID=HBCPDMXD016) |
| 11808 | Lactobacillus sp. strain 27-1 16S ribosomal RNA gene, partial sequence | 165 | 95% | 3.00E-37 | 98.91 | [KX499358.1](https://www.ncbi.nlm.nih.gov/nucleotide/KX499358.1?report=genbank&log$=nucltop&blast_rank=16&RID=HBCPDMXD016) |
| 11808 | Lactobacillus acidipiscis strain PR09 16S ribosomal RNA gene, partial sequence | 165 | 95% | 3.00E-37 | 98.91 | [KX139191.1](https://www.ncbi.nlm.nih.gov/nucleotide/KX139191.1?report=genbank&log$=nucltop&blast_rank=17&RID=HBCPDMXD016) |
| 11808 | Uncultured bacterium clone OTU_2465 16S ribosomal RNA gene, partial sequence | 165 | 95% | 3.00E-37 | 98.91 | [KU650871.1](https://www.ncbi.nlm.nih.gov/nucleotide/KU650871.1?report=genbank&log$=nucltop&blast_rank=18&RID=HBCPDMXD016) |
| 11808 | Lactobacillus sp. K30A 16S ribosomal RNA gene, partial sequence | 165 | 95% | 3.00E-37 | 98.91 | [KU714846.1](https://www.ncbi.nlm.nih.gov/nucleotide/KU714846.1?report=genbank&log$=nucltop&blast_rank=19&RID=HBCPDMXD016) |
| 11808 | Lactobacillus sp. K29C 16S ribosomal RNA gene, partial sequence | 165 | 95% | 3.00E-37 | 98.91 | [KU714845.1](https://www.ncbi.nlm.nih.gov/nucleotide/KU714845.1?report=genbank&log$=nucltop&blast_rank=20&RID=HBCPDMXD016) |
| 11808 | Lactobacillus acidipiscis gene for 16S ribosomal RNA, partial sequence, strain: SR7-1 | 165 | 95% | 3.00E-37 | 98.91 | [LC127171.1](https://www.ncbi.nlm.nih.gov/nucleotide/LC127171.1?report=genbank&log$=nucltop&blast_rank=21&RID=HBCPDMXD016) |
| 11808 | Lactobacillus acidipiscis partial 16S rRNA gene, strain MT19 | 165 | 95% | 3.00E-37 | 98.91 | [LN898271.1](https://www.ncbi.nlm.nih.gov/nucleotide/LN898271.1?report=genbank&log$=nucltop&blast_rank=22&RID=HBCPDMXD016) |
| 11808 | Lactobacillus sp. B4(2014) 16S ribosomal RNA gene, partial sequence | 165 | 95% | 3.00E-37 | 98.91 | [KM259929.1](https://www.ncbi.nlm.nih.gov/nucleotide/KM259929.1?report=genbank&log$=nucltop&blast_rank=23&RID=HBCPDMXD016) |
| 11808 | Uncultured prokaryote clone OTU100 16S ribosomal RNA gene, partial sequence | 165 | 95% | 3.00E-37 | 98.91 | [KF358873.1](https://www.ncbi.nlm.nih.gov/nucleotide/KF358873.1?report=genbank&log$=nucltop&blast_rank=24&RID=HBCPDMXD016) |
| 11808 | Uncultured Lactobacillus sp. gene for 16S ribosomal RNA, partial sequence, clone: 4X13 | 165 | 95% | 3.00E-37 | 98.91 | [LC002953.1](https://www.ncbi.nlm.nih.gov/nucleotide/LC002953.1?report=genbank&log$=nucltop&blast_rank=25&RID=HBCPDMXD016) |
| 11808 | Lactobacillus acidipiscis isolate ITA44 16S ribosomal RNA gene, partial sequence | 165 | 95% | 3.00E-37 | 98.91 | [KF297816.1](https://www.ncbi.nlm.nih.gov/nucleotide/KF297816.1?report=genbank&log$=nucltop&blast_rank=26&RID=HBCPDMXD016) |
| 11808 | Pediococcus sp. F3S1 16S ribosomal RNA gene, partial sequence | 165 | 95% | 3.00E-37 | 98.91 | [KF245543.1](https://www.ncbi.nlm.nih.gov/nucleotide/KF245543.1?report=genbank&log$=nucltop&blast_rank=27&RID=HBCPDMXD016) |
| 11808 | Uncultured Lactobacillus sp. clone 3c8 16S ribosomal RNA gene, partial sequence | 165 | 95% | 3.00E-37 | 98.91 | [KC755062.1](https://www.ncbi.nlm.nih.gov/nucleotide/KC755062.1?report=genbank&log$=nucltop&blast_rank=28&RID=HBCPDMXD016) |
| 11808 | Uncultured bacterium clone OTU93 16S ribosomal RNA gene, partial sequence | 165 | 95% | 3.00E-37 | 98.91 | [KC120652.1](https://www.ncbi.nlm.nih.gov/nucleotide/KC120652.1?report=genbank&log$=nucltop&blast_rank=29&RID=HBCPDMXD016) |
| 11808 | Lactobacillus acidipiscis gene for 16S rRNA, partial sequence, strain: Ni1465 | 165 | 95% | 3.00E-37 | 98.91 | [AB598991.1](https://www.ncbi.nlm.nih.gov/nucleotide/AB598991.1?report=genbank&log$=nucltop&blast_rank=30&RID=HBCPDMXD016) |
| 11808 | Lactobacillus acidipiscis gene for 16S rRNA, partial sequence, strain: Ni1462 | 165 | 95% | 3.00E-37 | 98.91 | [AB598989.1](https://www.ncbi.nlm.nih.gov/nucleotide/AB598989.1?report=genbank&log$=nucltop&blast_rank=31&RID=HBCPDMXD016) |
| 11808 | Lactobacillus acidipiscis gene for 16S rRNA, partial sequence, strain: Ni958 | 165 | 95% | 3.00E-37 | 98.91 | [AB598946.1](https://www.ncbi.nlm.nih.gov/nucleotide/AB598946.1?report=genbank&log$=nucltop&blast_rank=32&RID=HBCPDMXD016) |
| 11808 | Uncultured Lactobacillus sp. clone SCTB031 16S ribosomal RNA gene, partial sequence | 165 | 95% | 3.00E-37 | 98.91 | [JN650258.1](https://www.ncbi.nlm.nih.gov/nucleotide/JN650258.1?report=genbank&log$=nucltop&blast_rank=33&RID=HBCPDMXD016) |
| 11808 | Lactobacillus pobuzihii strain LZLJ22-3 16S ribosomal RNA gene, partial sequence | 165 | 95% | 3.00E-37 | 98.91 | [JQ043379.1](https://www.ncbi.nlm.nih.gov/nucleotide/JQ043379.1?report=genbank&log$=nucltop&blast_rank=34&RID=HBCPDMXD016) |
| 11808 | Lactobacillus similis strain LZLJ22-1 16S ribosomal RNA gene, partial sequence | 165 | 95% | 3.00E-37 | 98.91 | [JQ043378.1](https://www.ncbi.nlm.nih.gov/nucleotide/JQ043378.1?report=genbank&log$=nucltop&blast_rank=35&RID=HBCPDMXD016) |
| 11808 | Lactobacillus acidipiscis strain LZLJ19-3 16S ribosomal RNA gene, partial sequence | 165 | 95% | 3.00E-37 | 98.91 | [JQ043377.1](https://www.ncbi.nlm.nih.gov/nucleotide/JQ043377.1?report=genbank&log$=nucltop&blast_rank=36&RID=HBCPDMXD016) |
| 11808 | Lactobacillus acidipiscis strain LZLJ18-3 16S ribosomal RNA gene, partial sequence | 165 | 95% | 3.00E-37 | 98.91 | [JQ043376.1](https://www.ncbi.nlm.nih.gov/nucleotide/JQ043376.1?report=genbank&log$=nucltop&blast_rank=37&RID=HBCPDMXD016) |
| 11808 | Lactobacillus acidipiscis strain LZLJ12-3 16S ribosomal RNA gene, partial sequence | 165 | 95% | 3.00E-37 | 98.91 | [JQ043374.1](https://www.ncbi.nlm.nih.gov/nucleotide/JQ043374.1?report=genbank&log$=nucltop&blast_rank=38&RID=HBCPDMXD016) |
| 11808 | Lactobacillus acidipiscis strain LZLJ10-1 16S ribosomal RNA gene, partial sequence | 165 | 95% | 3.00E-37 | 98.91 | [JQ043372.1](https://www.ncbi.nlm.nih.gov/nucleotide/JQ043372.1?report=genbank&log$=nucltop&blast_rank=39&RID=HBCPDMXD016) |
| 11808 | Lactobacillus acidipiscis strain LZLJ8-3 16S ribosomal RNA gene, partial sequence | 165 | 95% | 3.00E-37 | 98.91 | [JQ043371.1](https://www.ncbi.nlm.nih.gov/nucleotide/JQ043371.1?report=genbank&log$=nucltop&blast_rank=40&RID=HBCPDMXD016) |
| 11808 | Lactobacillus acidipiscis strain LZLJ8-1 16S ribosomal RNA gene, partial sequence | 165 | 95% | 3.00E-37 | 98.91 | [JQ043370.1](https://www.ncbi.nlm.nih.gov/nucleotide/JQ043370.1?report=genbank&log$=nucltop&blast_rank=41&RID=HBCPDMXD016) |
| 11808 | Lactobacillus acidipiscis strain LZLJ4-3 16S ribosomal RNA gene, partial sequence | 165 | 95% | 3.00E-37 | 98.91 | [JQ043369.1](https://www.ncbi.nlm.nih.gov/nucleotide/JQ043369.1?report=genbank&log$=nucltop&blast_rank=42&RID=HBCPDMXD016) |
| 11808 | Lactobacillus acidipiscis strain LZLJ4-1 16S ribosomal RNA gene, partial sequence | 165 | 95% | 3.00E-37 | 98.91 | [JQ043368.1](https://www.ncbi.nlm.nih.gov/nucleotide/JQ043368.1?report=genbank&log$=nucltop&blast_rank=43&RID=HBCPDMXD016) |
| 11808 | Lactobacillus acidipiscis strain LZLJ2-3 16S ribosomal RNA gene, partial sequence | 165 | 95% | 3.00E-37 | 98.91 | [JQ043367.1](https://www.ncbi.nlm.nih.gov/nucleotide/JQ043367.1?report=genbank&log$=nucltop&blast_rank=44&RID=HBCPDMXD016) |
| 11808 | Lactobacillus sp. TRF8 16S ribosomal RNA gene, partial sequence | 165 | 95% | 3.00E-37 | 98.91 | [JN660076.1](https://www.ncbi.nlm.nih.gov/nucleotide/JN660076.1?report=genbank&log$=nucltop&blast_rank=45&RID=HBCPDMXD016) |
| 11808 | Lactobacillus sp. TRF5 16S ribosomal RNA gene, partial sequence | 165 | 95% | 3.00E-37 | 98.91 | [JN660074.1](https://www.ncbi.nlm.nih.gov/nucleotide/JN660074.1?report=genbank&log$=nucltop&blast_rank=46&RID=HBCPDMXD016) |
| 11808 | Lactobacillus sp. TRF7 16S ribosomal RNA gene, partial sequence | 165 | 95% | 3.00E-37 | 98.91 | [JN660059.1](https://www.ncbi.nlm.nih.gov/nucleotide/JN660059.1?report=genbank&log$=nucltop&blast_rank=47&RID=HBCPDMXD016) |
| 11808 | Lactobacillus sp. TTp14 16S ribosomal RNA gene, partial sequence | 165 | 95% | 3.00E-37 | 98.91 | [HQ141624.1](https://www.ncbi.nlm.nih.gov/nucleotide/HQ141624.1?report=genbank&log$=nucltop&blast_rank=48&RID=HBCPDMXD016) |
| 11808 | Lactobacillus sp. TTp13 16S ribosomal RNA gene, partial sequence | 165 | 95% | 3.00E-37 | 98.91 | [HQ141623.1](https://www.ncbi.nlm.nih.gov/nucleotide/HQ141623.1?report=genbank&log$=nucltop&blast_rank=49&RID=HBCPDMXD016) |
| 11808 | Lactobacillus sp. TTp12 16S ribosomal RNA gene, partial sequence | 165 | 95% | 3.00E-37 | 98.91 | [HQ141622.1](https://www.ncbi.nlm.nih.gov/nucleotide/HQ141622.1?report=genbank&log$=nucltop&blast_rank=50&RID=HBCPDMXD016) |
| 11808 | Lactobacillus sp. TTp6 16S ribosomal RNA gene, partial sequence | 165 | 95% | 3.00E-37 | 98.91 | [HQ141621.1](https://www.ncbi.nlm.nih.gov/nucleotide/HQ141621.1?report=genbank&log$=nucltop&blast_rank=51&RID=HBCPDMXD016) |
| 11808 | Lactobacillus sp. TTp4 16S ribosomal RNA gene, partial sequence | 165 | 95% | 3.00E-37 | 98.91 | [HQ141620.1](https://www.ncbi.nlm.nih.gov/nucleotide/HQ141620.1?report=genbank&log$=nucltop&blast_rank=52&RID=HBCPDMXD016) |
| 11808 | Lactobacillus sp. Tporo2 16S ribosomal RNA gene, partial sequence | 165 | 95% | 3.00E-37 | 98.91 | [HM534775.1](https://www.ncbi.nlm.nih.gov/nucleotide/HM534775.1?report=genbank&log$=nucltop&blast_rank=53&RID=HBCPDMXD016) |
| 11808 | Lactobacillus sp. MbBipro17 16S ribosomal RNA gene, partial sequence | 165 | 95% | 3.00E-37 | 98.91 | [HM534774.1](https://www.ncbi.nlm.nih.gov/nucleotide/HM534774.1?report=genbank&log$=nucltop&blast_rank=54&RID=HBCPDMXD016) |
| 11808 | Lactobacillus sp. Thmro2 16S ribosomal RNA gene, partial sequence | 165 | 95% | 3.00E-37 | 98.91 | [HM534773.1](https://www.ncbi.nlm.nih.gov/nucleotide/HM534773.1?report=genbank&log$=nucltop&blast_rank=55&RID=HBCPDMXD016) |
| 11808 | Lactobacillus acidipiscis strain 5-1 16S ribosomal RNA gene, partial sequence | 165 | 95% | 3.00E-37 | 98.91 | [HQ384296.1](https://www.ncbi.nlm.nih.gov/nucleotide/HQ384296.1?report=genbank&log$=nucltop&blast_rank=56&RID=HBCPDMXD016) |
| 11808 | Lactobacillus pobuzihii gene for 16S rRNA, partial sequence, strain: NBRC 103227 | 165 | 95% | 3.00E-37 | 98.91 | [AB326366.1](https://www.ncbi.nlm.nih.gov/nucleotide/AB326366.1?report=genbank&log$=nucltop&blast_rank=57&RID=HBCPDMXD016) |
| 11808 | Lactobacillus pobuzihii gene for 16S rRNA, partial sequence, strain: NBRC 103228 | 165 | 95% | 3.00E-37 | 98.91 | [AB326367.1](https://www.ncbi.nlm.nih.gov/nucleotide/AB326367.1?report=genbank&log$=nucltop&blast_rank=58&RID=HBCPDMXD016) |
| 11808 | Lactobacillus pobuzihii gene for 16S rRNA, partial sequence, strain: NBRC 103226 | 165 | 95% | 3.00E-37 | 98.91 | [AB326365.1](https://www.ncbi.nlm.nih.gov/nucleotide/AB326365.1?report=genbank&log$=nucltop&blast_rank=59&RID=HBCPDMXD016) |
| 11808 | Lactobacillus pobuzihii strain E100301 16S ribosomal RNA, partial sequence | 165 | 95% | 3.00E-37 | 98.91 | [NR_112694.1](https://www.ncbi.nlm.nih.gov/nucleotide/NR_112694.1?report=genbank&log$=nucltop&blast_rank=60&RID=HBCPDMXD016) |
| 11808 | Uncultured bacterium clone l_A23 16S ribosomal RNA gene, partial sequence | 165 | 95% | 3.00E-37 | 98.91 | [EU448891.1](https://www.ncbi.nlm.nih.gov/nucleotide/EU448891.1?report=genbank&log$=nucltop&blast_rank=61&RID=HBCPDMXD016) |
| 11808 | Lactobacillus acidipiscis gene for 16S rRNA, partial sequence, strain: NBRC 102164 | 165 | 95% | 3.00E-37 | 98.91 | [AB326357.1](https://www.ncbi.nlm.nih.gov/nucleotide/AB326357.1?report=genbank&log$=nucltop&blast_rank=62&RID=HBCPDMXD016) |
| 11808 | Uncultured compost bacterium clone 0B21 16S ribosomal RNA gene, partial sequence | 165 | 95% | 3.00E-37 | 98.91 | [DQ345474.1](https://www.ncbi.nlm.nih.gov/nucleotide/DQ345474.1?report=genbank&log$=nucltop&blast_rank=63&RID=HBCPDMXD016) |
| 11808 | Lactobacillus sp. YE06 16S ribosomal RNA gene, partial sequence | 165 | 95% | 3.00E-37 | 98.91 | [AF316585.1](https://www.ncbi.nlm.nih.gov/nucleotide/AF316585.1?report=genbank&log$=nucltop&blast_rank=64&RID=HBCPDMXD016) |
| 11808 | Lactobacillus sp. 121B 16S ribosomal RNA gene, partial sequence | 165 | 95% | 3.00E-37 | 98.91 | [AF305930.1](https://www.ncbi.nlm.nih.gov/nucleotide/AF305930.1?report=genbank&log$=nucltop&blast_rank=65&RID=HBCPDMXD016) |
| 11808 | Lactobacillus sp. LMK3 16S rRNA gene, strain LMK3 | 165 | 95% | 3.00E-37 | 98.91 | [AJ251560.1](https://www.ncbi.nlm.nih.gov/nucleotide/AJ251560.1?report=genbank&log$=nucltop&blast_rank=66&RID=HBCPDMXD016) |
| 11808 | Lactobacillus sp. FS1111 gene for 16S rRNA, partial sequence | 165 | 95% | 3.00E-37 | 98.91 | [AB023837.1](https://www.ncbi.nlm.nih.gov/nucleotide/AB023837.1?report=genbank&log$=nucltop&blast_rank=67&RID=HBCPDMXD016) |
| 11808 | Lactobacillus acidipiscis strain FS60-1 16S ribosomal RNA, partial sequence | 165 | 95% | 3.00E-37 | 98.91 | [NR_024718.1](https://www.ncbi.nlm.nih.gov/nucleotide/NR_024718.1?report=genbank&log$=nucltop&blast_rank=68&RID=HBCPDMXD016) |
| 11810 | Lactobacillus acidipiscis strain BP-1.2 16S ribosomal RNA gene, partial sequence | 165 | 95% | 3.00E-37 | 98.91 | [MF191678.1](https://www.ncbi.nlm.nih.gov/nucleotide/MF191678.1?report=genbank&log$=nucltop&blast_rank=1&RID=HBCRT9CB014) |
| 11810 | Uncultured Lactobacillus sp. clone xw3-167 16S ribosomal RNA gene, partial sequence | 165 | 95% | 3.00E-37 | 98.91 | [KU961720.1](https://www.ncbi.nlm.nih.gov/nucleotide/KU961720.1?report=genbank&log$=nucltop&blast_rank=2&RID=HBCRT9CB014) |
| 11810 | Lactobacillus acidipiscis strain ACA-DC 1533 genome assembly, chromosome: I | 165 | 95% | 3.00E-37 | 98.91 | [LT630287.1](https://www.ncbi.nlm.nih.gov/nucleotide/LT630287.1?report=genbank&log$=nucltop&blast_rank=3&RID=HBCRT9CB014) |
| 11810 | Uncultured Lactobacillus sp. clone SCTB037 16S ribosomal RNA gene, partial sequence | 165 | 95% | 3.00E-37 | 98.91 | [JN650260.1](https://www.ncbi.nlm.nih.gov/nucleotide/JN650260.1?report=genbank&log$=nucltop&blast_rank=4&RID=HBCRT9CB014) |
| 11810 | Lactobacillus sp. NBRC 107294 gene for 16S rRNA, partial sequence | 165 | 95% | 3.00E-37 | 98.91 | [AB682589.1](https://www.ncbi.nlm.nih.gov/nucleotide/AB682589.1?report=genbank&log$=nucltop&blast_rank=5&RID=HBCRT9CB014) |
| 11810 | Lactobacillus sp. NBRC 107255 gene for 16S rRNA, partial sequence | 165 | 95% | 3.00E-37 | 98.91 | [AB682553.1](https://www.ncbi.nlm.nih.gov/nucleotide/AB682553.1?report=genbank&log$=nucltop&blast_rank=6&RID=HBCRT9CB014) |
| 11810 | Uncultured compost bacterium clone 0B31 16S ribosomal RNA gene, partial sequence | 165 | 95% | 3.00E-37 | 98.91 | [DQ345484.1](https://www.ncbi.nlm.nih.gov/nucleotide/DQ345484.1?report=genbank&log$=nucltop&blast_rank=7&RID=HBCRT9CB014) |

| **Table S8. Effect of age on relative abundance of taxa: an ALDEx2 analysis. Positive values indicate taxa was more common among children. Stimulated saliva samples were collected from 61 individuals from the Center for Oral Health Research in Appalachia Study. Species in bold indicate taxa are also significant for 1 or more metals**. | |
| --- | --- |
| **Taxa** | **Effect Size** |
| *Acinetobacter_haemolyticus* | -1.17 |
| *Neisseria_flava mucosa pharyngis* | -0.89 |
| *unclassified Clostridiales C_sulci infirmum* | -0.87 |
| *Porphyromonas_HF001* | -0.80 |
| *Pasteurellaceae_f_12032* | -0.77 |
| *Kingella_denitrificans* | -0.74 |
| *Gemella_haemolysans* | -0.72 |
| *Alloprevotella_IK062* | -0.71 |
| *Aggregatibacter_aprophilus* | -0.71 |
| *Capnocytophaga_sputigena* | -0.70 |
| *Aggregatibacter_segnis* | -0.69 |
| *Porphyromonas_catoniae* | -0.67 |
| *Porphyromonas_CW034* | -0.67 |
| *Pasteurellaceae_f_25134* | -0.62 |
| ***Granulicatella_elegans*** | -0.59 |
| ***Neisseria_meningitidis polysaccharea*** | -0.59 |
| *Prevotella_oral taxon 299* | -0.56 |
| *Granulicatella_sp_9748* | -0.55 |
| *Neisseria_bacilliformis* | -0.54 |
| *Cardiobacterium_hominis* | -0.54 |
| *Neisseria_sp_16970* | -0.53 |
| *Solobacterium_moorei* | -0.53 |
| *Kingella_oralis* | -0.52 |
| *Haemophilus_parainfluenzae* | -0.51 |
| *Neisseria_sp_17146* | -0.49 |
| *Haemophilus_sp_19211* | -0.48 |
| *Rothia_aeria* | -0.48 |
| *Leptotrichia_sp_6067* | -0.47 |
| ***Abiotrophia_defectiva*** | -0.44 |
| *Haemophilus_sp_18674* | -0.44 |
| *Lautropia_mirabilis* | -0.44 |
| *Haemophilus_sp_19213* | -0.44 |
| *Bergeyella_JF233961* | -0.44 |
| *Neisseria_AP085* | -0.40 |
| *Moraxella_caprae* | -0.38 |
| *Veillonella_59q* | 0.30 |
| ***Lactobacillus_sp_11809*** | 0.38 |
| *Streptococcus_sp_18176* | 0.38 |
| *Actinomyces_viscosus naeslundii oris* | 0.38 |
| *Streptococcus_peroris* | 0.41 |
| ***Lactobacillus_sp_11800*** | 0.42 |
| ***Streptococcus_GU045364*** | 0.43 |
| ***Lactobacillus_sp_11798*** | 0.45 |
| *Lactobacillus_fermentum* | 0.47 |
| *Prevotella_sp_9410* | 0.47 |
| ***Prevotella_sp_9409*** | 0.49 |
| *Prevotella_sp_9398* | 0.49 |
| ***Prevotella_sp_9421*** | 0.49 |
| *Prevotella_sp_9402* | 0.51 |
| *Streptococcus_sp_18830* | 0.57 |
| *Atopobium_rimae* | 0.58 |
| *Fusobacterium_nucleatum subsp. fusiforme* | 0.63 |
| *Streptococcus_sp_18846* | 0.64 |
| The effect size comparing those in the 18+ age group to those in the 7-17 age group for each species is the difference between groups relative to an estimate of within-group dispersion. Only associations with Benjamini-Hochberg corrected p-values <0.1 are shown. | |

| **Table S9. Odds ratios for presence of decayed teeth by metal tertile of essential minerals, other heavy metals, and metals with some evidence of physiologic function, adjusted for age and income.** | | |
| --- | --- | --- |
| **Metal** | **High vs low** | **Medium vs low** |
| Antimony | 2.80 ( 0.58, 13.50) | 3.74 ( 0.81, 17.18) |
| Arsenic | 1.09 ( 0.28, 4.23) | 0.69 ( 0.12, 3.99) |
| Barium | 1.10 ( 0.23, 5.21) | 2.34 ( 0.43, 12.59) |
| Beryllium | 0.15 ( 0.02, 1.20) | NA |
| Cadmium | 0.54 ( 0.12, 2.38) | 1.13 ( 0.23, 5.49) |
| Cesium | 0.88 ( 0.17, 4.49) | 1.06 ( 0.22, 5.11) |
| Chromium | 1.77 ( 0.35, 9.06) | 0.72 ( 0.17, 3.12) |
| Cobalt | 1.73 ( 0.37, 8.04) | 0.90 ( 0.21, 3.86) |
| Copper | 0.29 ( 0.05, 1.74) | 0.18 ( 0.03, 1.01) |
| Lead | 1.15 ( 0.24, 5.48) | 1.76 ( 0.38, 8.25) |
| Manganese | 1.65 ( 0.38, 7.19) | 2.02 ( 0.45, 9.12) |
| Mercury | 3.00 ( 0.55, 16.40) | 0.72 ( 0.17, 3.00) |
| Molybdenum | 2.18 ( 0.49, 9.61) | 1.57 ( 0.33, 7.40) |
| Nickel | 0.44 ( 0.09, 2.16) | 1.97 ( 0.41, 9.45) |
| Platinum | 0.43 ( 0.02, 8.04) | NA |
| Thallium | 0.14 ( 0.01, 1.67) | NA |
| Tin | 4.78 ( 0.89, 25.76) | 1.64 ( 0.39, 6.96) |
| Tungsten | 1.55 ( 0.28, 8.68) | 0.73 ( 0.17, 3.18) |
| Uranium | 0.38 ( 0.08, 1.81) | NA |
| Vanadium | 1.35 ( 0.30, 6.03) | 1.83 ( 0.41, 8.29) |
| Zinc | 0.94 ( 0.21, 4.18) | 1.89 ( 0.40, 8.93) |

Figure S1. Microbiome-wide associations of selected metals with salivary taxa. If an oligotype was determined that could not be resolved to species level the oligotype is indicated by a number. The line indicates a p-value <0.1 after Benjamini-Hochberg correction. Blue circles indicated decreased abundance with higher levels of the metal, and gold circles indicated increased abundance with higher levels of the metal. 1SA. arsenic, 1SB. antimony, and 1SC. mercury. Saliva samples were collected from 61 adults and children participating in the Center for Oral Health Research in Appalachia cohort 2 (COHRA 2)


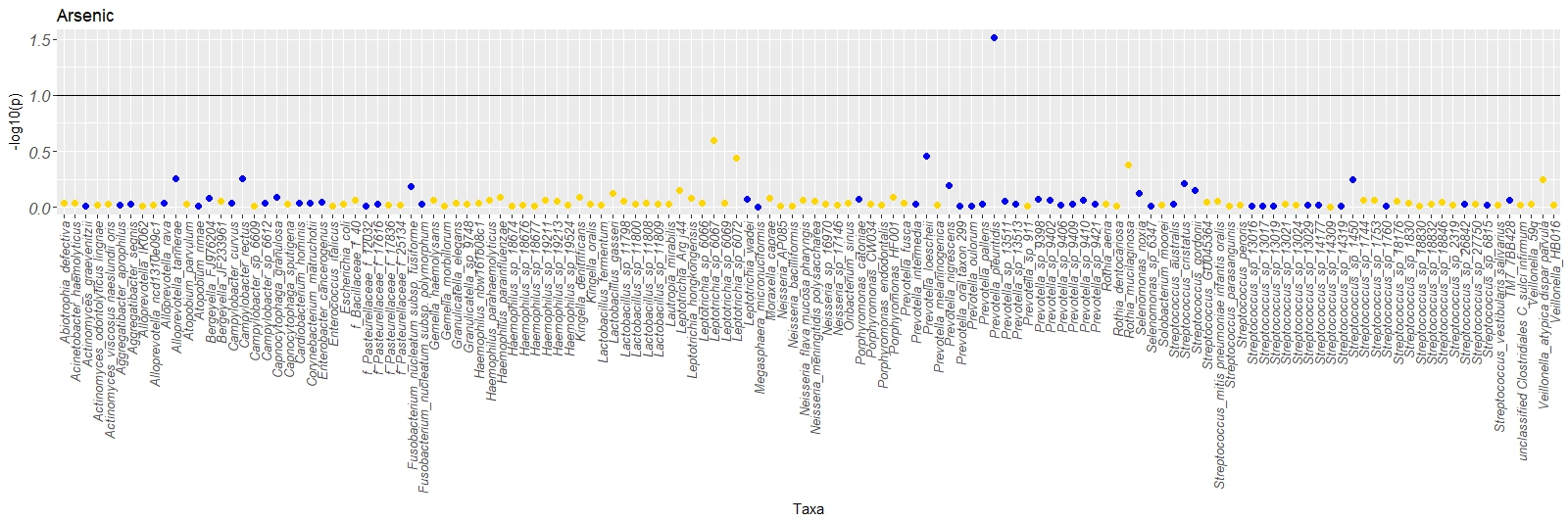

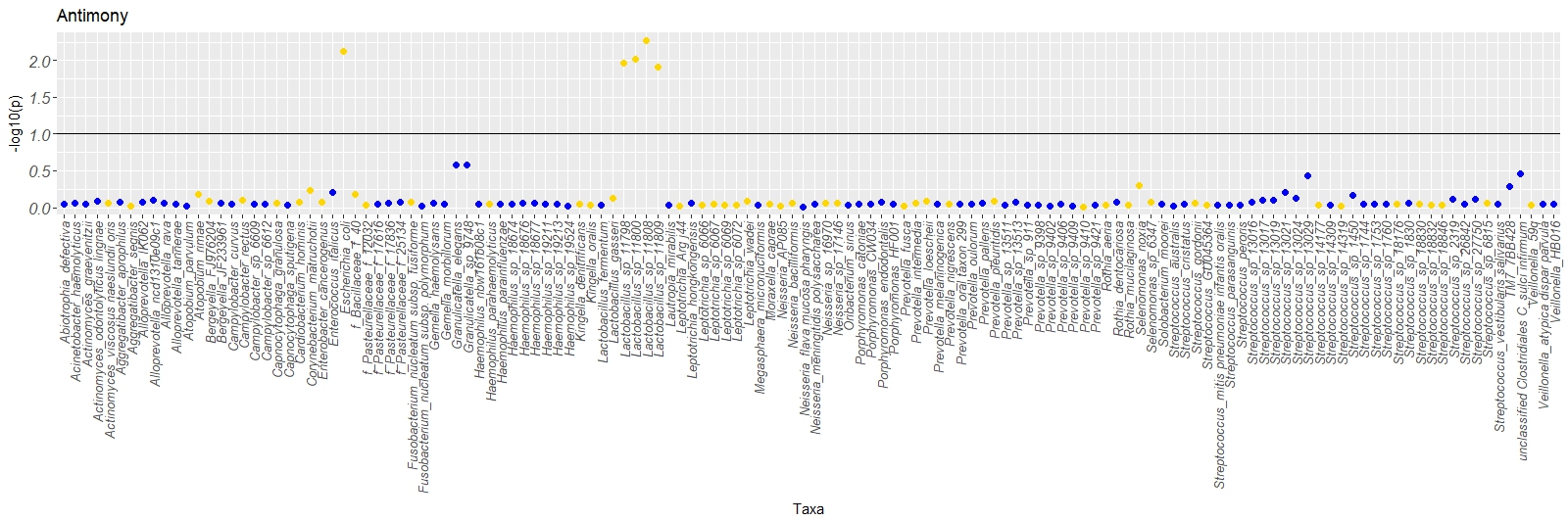
Figure S1A. Figure S1B.


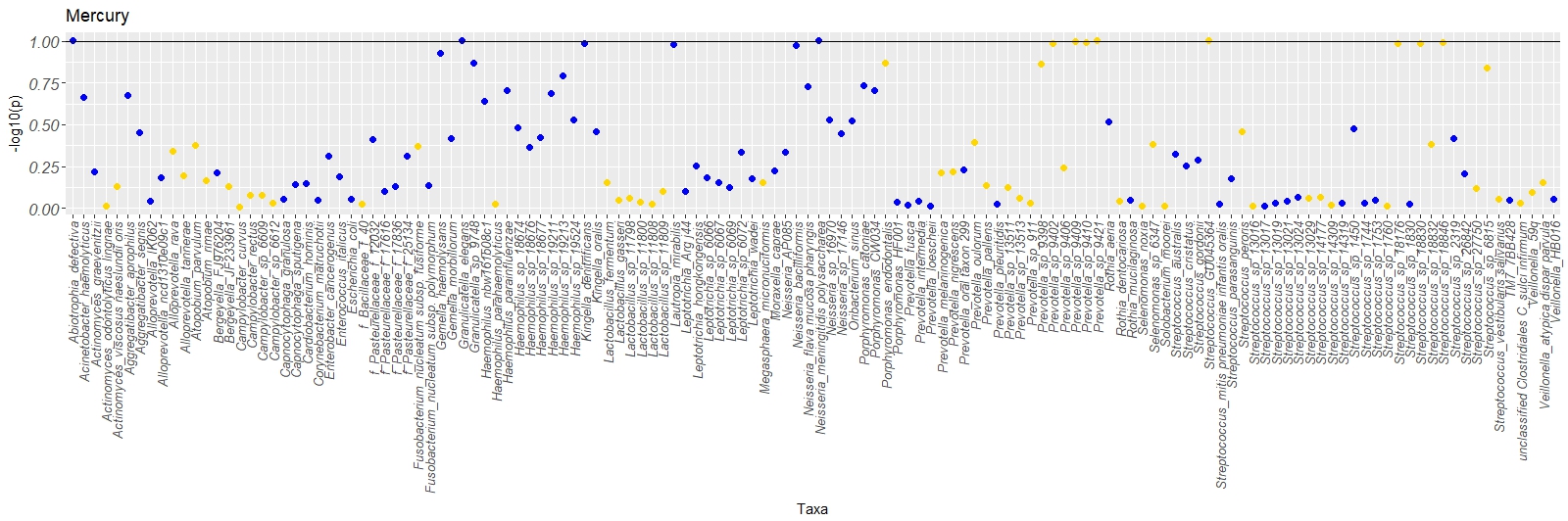


Figure S1C.

**References for Supplemental Material**

61. Royal Society of Chemistry (2019). Periodic Table. Last accessed on 4/29/2019. Available at: http://www.rsc.org/periodic-table/.

62. Agency for Toxic Substances & Disease Registry. ATSDR(2004). Public Health Statement for Cobalt. Last accessed on 4/29/2019. Accessible at: <https://www.atsdr.cdc.gov/PHS/PHS.asp?id=371&tid=64>

63. Medline Plus. U.S. National Library of Medicine (2019). Copper in diet. Last accessed on 4/29/2019. Accessible at: [https://medlineplus.gov/ency/article/002419.htm (2019](https://medlineplus.gov/ency/article/002419.htm%20(2019)

64. Agency for Toxic Substances & Disease Registry. ATSDR (2017). Public Health Statement for Molybdenum. Last accessed on 4/29/2019. PDF available at: <https://www.atsdr.cdc.gov/toxprofiles/tp212-c1.pdf>

65. Agency for Toxic Substances & Disease Registry. ATSDR (2017). Public Health Statement for Antimony. Last accessed on 4/29/2019. Available at: <https://www.atsdr.cdc.gov/phs/phs.asp?id=330&tid=58>

66. Agency for Toxic Substances & Disease Registry. ATSDR (2007). Public Health Statement for Arsenic. Last accessed on 4/29/2019. Available at: <https://www.atsdr.cdc.gov/phs/phs.asp?id=18&tid=3>

67. Agency for Toxic Substances & Disease Registry. ATSDR (2007). Public Health Statement for Barium. Last accessed on 4/29/2019. Available at:<https://www.atsdr.cdc.gov/phs/phs.asp?id=325&tid=57>

68. Agency for Toxic Substances & Disease Registry. ATSDR (2002). Public Health Statement for Beryllium. Last accessed on 4/29/2019. Available at: <https://www.atsdr.cdc.gov/phs/phs.asp?id=339&tid=33>

69. Agency for Toxic Substances & Disease Registry. ATSDR (2012). Public Health Statement for Cadmium. Last accessed on 4/29/2019. Available at: <https://www.atsdr.cdc.gov/phs/phs.asp?id=46&tid=15>

70. Delaware Health and Social Services. Division of Public Health (2015). Cesium. Last accessed on 4/29/2019. PDF Available at: <https://dhss.delaware.gov/dhss/dph/files/cesiumfaq.pdf>

71. Agency for Toxic Substances & Disease Registry. ATSDR (2007). Public Health Statement for Lead. Last accessed on 4/29/2019. Available at: <https://www.atsdr.cdc.gov/phs/phs.asp?id=92&tid=22>

72. Orecchio, S., & Amorello, D. (2019). Platinum and Rhodium in Potato Samples by Using Voltammetric Techniques. *Foods (Basel, Switzerland)*, ***8*(2),** 59. doi:10.3390/foods8020059

73. Agency for Toxic Substances & Disease Registry. ATSDR (1992). Public Health Statement for Thallium. Last accessed on 4/29/2019. Available at: <https://www.atsdr.cdc.gov/phs/phs.asp?id=307&tid=49>

74. Agency for Toxic Substances & Disease Registry. ATSDR (2005). Public Health Statement for Tin. Available at: <https://www.atsdr.cdc.gov/phs/phs.asp?id=541&tid=98>

75. Agency for Toxic Substances & Disease Registry. ATSDR (2013). Public Health Statement for Uranium. Last accessed on 4/29/2019. Available at: <https://www.atsdr.cdc.gov/phs/phs.asp?id=438&tid=77>

76. Agency for Toxic Substances & Disease Registry. ATSDR (2015). Public Health Statement for Chromium. Last accessed on 4/29/2019. Available at: <https://www.atsdr.cdc.gov/phs/phs.asp?id=60&tid=17>

77. Agency for Toxic Substances & Disease Registry. ATSDR (2005). Public Health Statement for Nickel. Last accessed on 4/29/2019. Available at: <https://www.atsdr.cdc.gov/phs/phs.asp?id=243&tid=44>

78. Agency for Toxic Substances & Disease Registry. ATSDR (2011). Public Health Statement for Tungsten. Last accessed on 4/29/2019. Available at: <https://www.atsdr.cdc.gov/phs/phs.asp?id=804&tid=157>

79. Agency for Toxic Substances & Disease Registry. ATSDR (2012). Public Health Statement for Vanadium. Last accessed on 4/29/2019. Available at: <https://www.atsdr.cdc.gov/phs/phs.asp?id=274&tid=50>
